# Rapid lung ventilation MRI using parahydrogen-induced polarization of propane gas†

**DOI:** 10.1039/d4an01029a

**Published:** 2024-11-12

**Authors:** Md Raduanul H. Chowdhury, Clementinah Oladun, Nuwandi M. Ariyasingha, Anna Samoilenko, Tarek Bawardi, Dudari B. Burueva, Oleg G. Salnikov, Larisa M. Kovtunova, Valerii I. Bukhtiyarov, Zhongjie Shi, Kehuan Luo, Sidhartha Tan, Juri G. Gelovani, Igor V. Koptyug, Boyd M. Goodson, Eduard Y. Chekmenev

**Affiliations:** a Department of Chemistry, Integrative Biosciences (Ibio), Wayne State University, Karmanos Cancer Institute (KCI) Detroit Michigan 48202 USA chekmenevlab@gmail.com; b International Tomography Center, SB RAS 3A Institutskaya St. Novosibirsk 630090 Russia; c Boreskov Institute of Catalysis SB RAS 5 Acad. Lavrentiev Pr. Novosibirsk 630090 Russia; d Department of Pediatrics, Wayne State University Detroit Michigan 48202 USA; e United Arab Emirates University Al Ain United Arab Emirates; f School of Chemical & Biomolecular Sciences, Materials Technology Center, Southern Illinois University Carbondale IL 62901 USA

## Abstract

Proton-hyperpolarized contrast agents are attractive because they can be imaged on virtually any clinical MRI scanner, which is typically equipped to scan only protons rather than heteronuclei (*i.e.*, anything besides protons, *e.g.*, ^13^C, ^15^N, ^129^Xe, ^23^Na, *etc*.). Even though the lifetime of the proton spin hyperpolarization is only a few seconds, it is sufficient for inhalation and scanning of proton-hyperpolarized gas media. We demonstrate the utility of producing hyperpolarized propane gas *via* heterogeneous parahydrogen-induced polarization for the purpose of ventilation imaging in an excised rabbit lung model. The magnetization of protons in hyperpolarized propane gas is similar to that of tissue water protons, making it possible to rapidly perform lung ventilation imaging with a 0.35 T clinical MRI scanner. Here, we demonstrate the feasibility of rapid (2 s) lung ventilation MRI in excised rabbit lungs using hyperpolarized propane gas with a 1 × 1 mm^2^ pixel size using a 50 mm slice thickness, and a 1.7 × 1.7 mm^2^ pixel size using a 9 mm slice thickness.

## Abstract 2

The feasibility of ultrafast (1.7 s) ventilation MRI with a 1 × 1 × 50 mm^3^ voxel size is demonstrated using hyperpolarized propane gas contrast agent in excised rabbit lungs on a 0.35 T clinical MRI scanner without any scanner modification.

## Graphical abstract

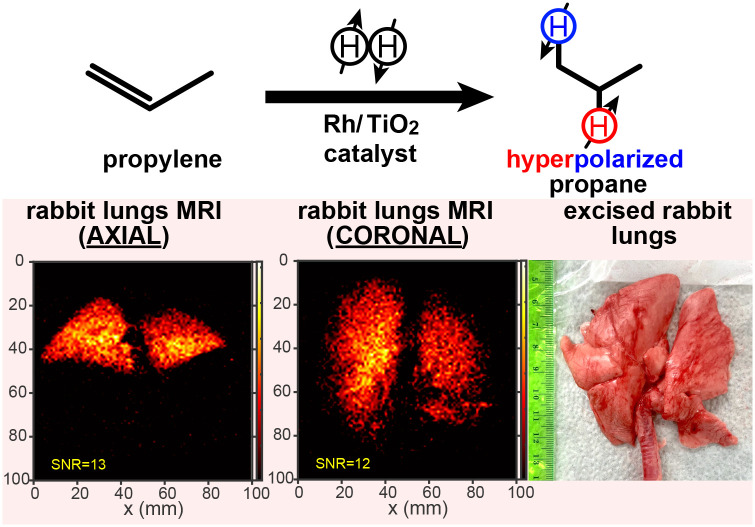

## Introduction

In the presence of a magnetic field, nuclear spins gain population differences among their Zeeman energy levels, giving rise to a net magnetization of a given nuclear spin ensemble. The signal detected in NMR and its sister technique MRI is directly proportional to the net magnetization. In turn, the nuclear spin magnetization is directly proportional to the concentration of nuclear spins and the population difference of adjacent energy levels (termed spin polarization *P*).^1^ Therefore, the NMR signal scales linearly with *P*. Although *P* scales linearly with the applied magnetic field *B*_0_ (main static magnetic field for the NMR magnet or MRI scanner) under conditions of vanishingly small *P* values, nuclear spin polarization remains low even within the strong magnetic fields of clinical MRI scanners (*e.g.*, *P* = 10^−5^ at 3 T for ^1^H nuclei).^2^ As a result of this fundamental limitation, tissues become only slightly magnetized, which restricts most clinical MR imaging to water (and fat) protons with a high overall concentration of ∼80 M.

NMR hyperpolarization temporarily increases *P* by several orders of magnitude (in some cases order unity^3–5^), rendering correspondingly massive gains in spin magnetization and MRI signals.^2,6–8^ Hyperpolarized (HP) MRI is emerging as a safe molecular imaging technique because it relies on harmless, non-ionizing radiofrequency (RF) waves for detection.^9–14^ Indeed, the first HP MRI contrast agent, hyperpolarized ^129^Xe gas, was recently approved by the FDA for lung ventilation imaging to diagnose a wide range of pulmonary diseases. The resonance frequency of ^129^Xe gas is approximately 3.6 times lower than that of proton spins; thus, ^129^Xe lung images are naturally free from tissue background signals, making image interpretation streamlined and straightforward.^14–20^ This important feature of HP ^129^Xe represents a double-edged sword because the dominating majority (over 99.5%) of clinical MRI scanners are set up to record images of protons only. Since clinical MRI scanners employ narrow-band electronics, they cannot readily perform a HP ^129^Xe MRI scan because ^129^Xe resonates at a vastly different (∼3.6 times lower) frequency. Although, in principle, a clinical MRI scanner can be upgraded with a multi-nuclear capability for HP ^129^Xe scanning, such upgrades are complex and expensive (over $0.5 M). Moreover, while all the leading vendors of clinical MRI scanners (*i.e.*, Siemens, GE Healthcare and Philips) offer multi-nuclear capability, it is typically limited to their 3.0 T MRI scanners, which is a downside since 3.0 T MRI scanners are inherently more expensive compared to lower-field MRI scanners (furthermore, the owners of most lower-field MRI scanners are effectively locked out of the opportunity for multi-nuclear capability). Moreover, the production of HP ^129^Xe is slow (it can take ∼0.5 hours per dose) and expensive (the hyperpolarizer device cost is >$0.3 M).^21–28^ Although continuous-flow Xe hyperpolarizers perform the process of ^129^Xe hyperpolarization continuously, the continuous-flow production process operates with a lean Xe mixture (typically 1–2% with the remaining fraction of buffering He gas).^21^ HP Xe is cryogenically frozen in such systems during the production over the course of 30–60 minutes.^21^ So, although such a hyperpolarization process employs continuous flow, a batch of HP Xe is actually produced *via* the process of thawing of frozen Xe (following 30–60 minutes of accumulation) to maximize the concentration of HP ^129^Xe in the produced gas contrast agent and the resulting MRI signal after *in vivo* administration.^21^ These limitations of HP ^129^Xe pose substantial translational challenges for making this life-saving technology broadly accessible.

One way to address this limitation is to deploy proton-HP contrast agents.^29,30^ For example, HP water was proposed.^31^ However, proton *T*_1_ is typically 1–2 seconds *in vivo*, making the detection window (usually defined as several *T*_1_ values, where *T*_1_ is the exponential decay constant) insufficiently small compared to that of blood circulation (over 20 s); therefore, making the practical utility of this otherwise simple and streamlined technology possible is challenging in the clinical context.

However, unlike an intravenous injection of an HP contrast agent, requiring blood circulation for delivery to an organ of interest, inhalation of the HP contrast agent into the lungs can be accomplished quickly, *i.e.*, on a time scale comparable to proton *T*_1_ or faster.^32,33^ Indeed, the feasibility of production of proton-hyperpolarized gases has been demonstrated for MRI applications in catalysis.^30,34^ Moreover, the more recent feasibility demonstrations of clinical-scale production of proton-HP gases have rekindled interest in exploring their clinical utility as gas contrast media.^7,8,33,35–41^ In this paper, we employed proton-HP propane gas to demonstrate that high-resolution (1 × 1 mm^2^ pixel size using 50 mm slice thickness) rapid ventilation lung MRI is feasible on a low-field clinical scanner.

## Materials and methods

**Fig. 1:**
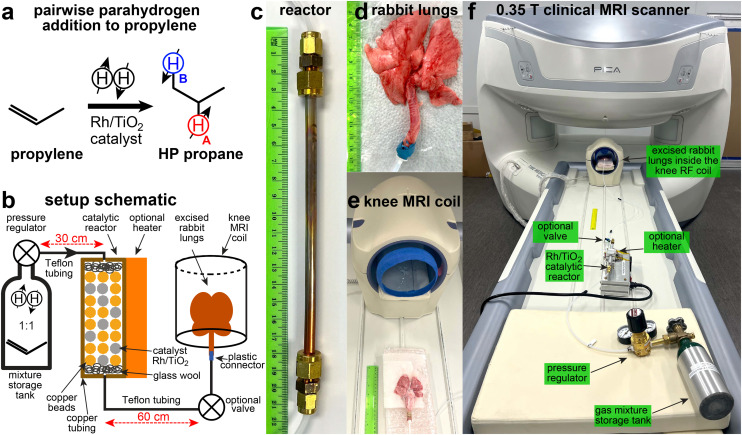
(a) Schematic of pairwise p-H_2_ addition to propylene to produce HP propane gas. (b) Schematic of the experimental setup showing the gas flow path through the catalytic reactor into excised rabbit lungs connected *via* Teflon tubing. (c) Close-up photograph of the catalytic reactor. (d) Photograph of representative excised rabbit lungs connected to a Teflon tubing. (e) Photograph of a knee MRI coil and the excised rabbit lung before positioning them inside the RF coil. (f) Annotated photograph of the experimental setup for conducting ventilation MRI imaging of excised rabbit lungs, utilizing a clinical knee RF coil, a tank with a gas mixture, and a catalytic reactor with a Rh/TiO_2_ catalyst. During the experiment, the lungs reside at the homogeneous field of the MRI scanner (0.35 T), the reactor resides at an estimated field of less than 1 mT, and the mixture storage tank resides at a magnetic field of less than 0.1 mT. The MRI scanner was shimmed using a brain phantom (placed inside the brain MRI coil) provided by the vendor prior to experimentations with HP propane gas and excised rabbit lungs.

The HP propane gas employed in this study was produced *via* parahydrogen-induced polarization (PHIP) technique^42,43^ (Fig. 1a). Briefly, parahydrogen (p-H_2_, over 95% para-state;^44^ 99.999% purity, Airgas) was mixed with propylene gas (>99% purity, Airgas, PP CP35) in a 1 : 1 ratio inside a high-pressure 0.7-liter aluminum storage tank. The produced mixture (10 bar pressure) was directed through a high-flow (0.15 standard liter per second (sLs) flow rate) reactor, containing a heterogeneous^45,46^ Rh/TiO_2_ catalyst (0.2 g, 0.9 wt% Rh content^47^), placed inside a 1/4′′ outer-diameter (OD) copper tubing (Fig. 1b). The reactant gas mixture was directed *via* Teflon tubing (1/8′′ OD, 1/16′′ inner diameter) through the catalytic reactor (*T* > 100 °C as a result of exothermic reaction), Fig. 1c. The temperature of the reactor was achieved by a combination of self-heating through the process of the exothermic reactor or applying heat to the reactor using a heat gun or an optional heater (both approaches yielded similar results in our studies). The reactor resided outside the main field of the MRI scanner at an estimated magnetic field below 1 mT. Pairwise p-H_2_ addition to propylene results in the symmetry breaking of nascent p-H_2_ hydrogens, thus, converting the singlet spin order to the HP states of H_A_ and H_B_ sites (denoted by emphasizing colors in Fig. 1a). The estimated *P*_H_ of HP protons H_A_ and H_B_ in the produced propane gas was 0.5–1% at an estimated 40 mM gas-phase concentration of produced HP propane at 1 atm.^36^ At 1% proton polarization, the anticipated polarization and signal enhancement is 8000 fold at 0.35 T, making it an equivalent to a 320 M thermally polarized proton species. Thus, the potential contrasting signal from HP gas (*P*_H_ = 1%) is anticipated to surpass that of water protons (*ca*. 80 M proton concentration *in vivo*) by several fold. In practice, the maximum attainable HP gas signal in the experiments reported here is attenuated by two primary effects. First, the HP state decays during the propane delivery from the reactor to the excised lungs. Second, the experiments are performed at 0.35 T, where partial cancellation of H_A_ and H_B_ resonances (with a chemical shift difference of 0.45 ppm (or ∼7 Hz) and a 180° phase difference with respect to each other^48,49^) is anticipated as *J*_H_A_–H_B__ (∼7.2 Hz) and the corresponding septet of H_A_ and triplet of H_B_ will have substantial spectral overlap and potential signal cancelation as H_A_ and H_B_ have an opposite phase.

The excised rabbit lungs were obtained from animals that were euthanized under unrelated animal studies (Wayne State University IACUC approval # IACUC-23-01-5364).^50–53,73–75^ Following the lung excision from a freshly euthanized animal, the lungs were transferred to the MRI scanner facility (∼15 min) and connected to the Teflon tubing line using plastic adapting connectors and a rubber band. Next, the lungs were inflated with air using a tire pump, and placed under water to confirm that no substantial gas leaks were present. “Leaky” lungs were discarded (in approximately 50% cases), while the HP gas studies proceeded with non-“leaky” lungs. Following the placement of the lungs on a wetted bedding (to ensure that lungs were maintained wet), they were placed inside a 3.6-liter empty plastic container that was fitted inside the clinical knee detection RF coil provided by the vendor (Time Medical) of the 0.35 T clinical MRI. The plastic enclosure was utilized to prevent excessive drying of the lungs’ tissue during experiments. The lungs were sprayed with water approximately every 0.5 hours to maintain their integrity. Overall, it was possible to utilize a set of intact lungs for 20–40 gas injections throughout a day of experiments. At the end of the day, the lungs were placed in the refrigerator and maintained at 5 °C. It was possible to re-use the excised lungs for an additional day following the removal from the refrigerator.

The HP propane gas exiting from the reactor was directed to the excised rabbit lungs. Following lung inflation with HP gas (1–1.5 s) with an estimated volume of 0.1–0.2 standard liters (sL), the gas flow was stopped.

A rapid 2D gradient echo (GRE) sequence was composed of 16 repeat back-to-back scans with a total scan time 1.7 s for each scan with a 96 × 96 imaging matrix. Other MR imaging parameters used in these experiments were 100 × 100 mm^2^ field of view (FOV), 30° slice-selective excitation RF pulse, 10.42 kHz spectral width, 17.8 ms and 20.27 ms repetition time (TR), and 8.5 ms and 9.75 ms echo time (TE), respectively. A separate (lower-resolution) MR scanning protocol was also utilized with a reduced imaging matrix to 64 × 64, resulting in the reduction of each scan from 1.7 s to 0.94 s with corresponding values of TR of 14.7 ms and TE of 6.97 ms.

The imaging sequence was initiated a few seconds before the administration of HP gas into the lungs to ensure the HP propane gas ventilation would be recorded immediately upon gas delivery into the lungs (with the goal of minimizing polarization losses due to *T*_1_ decay of the HP state). The sequence employed a slice thickness of 50 mm, effectively resulting in 2D projection imaging. Additional images with smaller slice thickness (*e.g.*, 9 mm) were also acquired albeit at lower spatial resolution. Typically, the HP signal persisted for 1–2 scans due to rapid depolarization by 30° RF excitation pulses, but longer acquisitions can be made in principle to extend the scanning time window. The imaging data were additionally post-processed *via* custom-written MATLAB code to perform subtraction of the background signal from the thermally polarized excised lung tissue and the surrounding wet blanket (ESI†). For background signal subtraction, a difference image was computed between the image with inflated lungs containing HP gas (usually, scan #4 to #5) and an image acquired after inflation and HP state depolarization (typically, scan #5 or #6). Additionally, the mean SNR is calculated by dividing the average intensity of signal pixel by the root mean square (RMS) value of the noise obtained from a 8 × 8 matrix in the noise region (see the ESI† for examples).

## Results and discussion

**Fig. 2:**
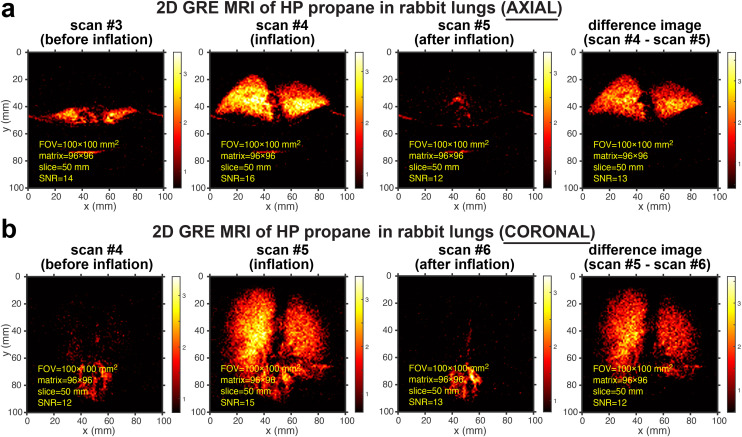
Rapid slice-selective 2D GRE images of HP propane gas rapidly expanding in excised rabbit lungs, acquired by utilizing a 0.35 T MRI scanner and a knee RF coil. (a) Axial projection of the excised rabbit lungs recorded before inflation (scan #4), during inflation (scan #5), and after inflation (scan #6) with HP propane gas at 1.7 s temporal resolution. (b) Corresponding scans from coronal projection. The difference images for both axial and coronal projections were obtained as the difference between scan #5 and scan #6 (Fig. S22 and S23† show the screenshots of the MRI scans as visualized with a 0.35 T scanner without any additional data processing). The corresponding mean SNR values associated with each image were obtained as described in detail in the ESI.† The axial and coronal scans were acquired with a 100 × 100 mm^2^ FOV, a 50 mm slice thickness, a 30° RF excitation pulse, a 96 × 96 imaging matrix, and post-processing interpolation to 768 × 768 pixels. A total of 16 repeat scans were recorded in 27 s, with a repetition time (TR) of 17.8 ms and an echo time (TE) of 8.55 ms.

Fig. 2 highlights the highest-resolution images that were obtained in this study. The excised rabbit lungs were ventilated and visualized with HP propane gas in the axial (Fig. 2a) and coronal projections (Fig. 2b). These images demonstrate good spatial resolution of lung ventilation MRI using HP propane gas. Indeed, Fig. 2a and b show high-resolution images with a pixel size of 1 × 1 mm^2^ acquired using a field of view (FOV) of 100 × 100 mm^2^ and an imaging matrix size of 96 × 96 (with a slice thickness of 50 mm), with a mean SNR of 12–13 in the background-subtracted images. Each imaging series (Fig. 2a and b, respectively) shows the selected repeat scans over the same anatomical location: as the HP propane gas reaches the lungs, the image denoted as “inflation” exhibits the HP gas signals throughout the lungs in addition to the background signal from thermally polarized tissues. In most cases, the signal from HP gas persisted only in one image (additional data analysis for Fig. 2b is shown in Fig. S27†). However, in cases where a higher SNR was available (*e.g.*, Fig. S13a†), the lung ventilation visualization was seen for more than one scan (in those cases, the thermally polarized background image subtraction procedure employed the post-inflation image, where the HP state was deemed to be depolarized rather than employed the scan acquired immediately “after inflation”). The image denoted as “before inflation” corresponds to the image acquired from thermally polarized samples of the collapsed lungs (before gas injection) and the surrounding wet blanket. MRI scanning with HP propane was reproducible: the comparative repeat scans are shown in Fig. S1.† The feasibility of achieving high spatial resolution is important because better-quality images allow the detection of smaller ventilation defects, *i.e.*, potentially enabling earlier diagnosis of lung diseases. Moreover, the recent studies with HP propane gas injected in excised pig lungs allowed achieving only a 4 × 4 mm^2^ pixel size (using the same slice thickness and a similar MRI scanner setup) corresponding to 15-times lower spatial resolution.^36^ The more-than-order-of-magnitude lower image resolution in the previous study is likely due to an overall slower inflation process with HP gas, rendering substantially greater depolarization losses in the previous study. Specifically, the inflation of the pig lungs with HP propane gas (0.7 sL) was performed over 5 seconds in the previous study,^36^ and over 95% of HP state magnetization was likely lost due to a fast decay constant (*T*_S_ was estimated between 1 and 2 s; *T*_S_ refers to the mono-exponential decay of long-lived spin states (LLSS),^32,33,54,55^ which are known to exist at low magnetic fields for HP propane gas produced under ALTADENA conditions^49^): work is in progress to perform precise mapping of HP state decay rates in excised rabbit lungs at 0.35 T. In this work, the rabbit lungs (injected gas volume was estimated at 0.1–0.2 sL) were injected in 1–1.5 s, *i.e.*, approximately 5–7 times faster than in the previous experiments. While HP state depolarization losses have occurred in the experiments with the rabbit lungs during the gas administration too, these losses were likely at least an order of magnitude lower compared to the previous experiments with larger pig lungs;^36^ we note that developing a more quantitative model of HP state polarization losses during gas delivery and imaging requires the quantitative knowledge of the HP state decay constant, which is not available to us at the moment (although active experiments are in progress and will be reported in the future). This fact underscores the need for high-capacity on-demand production of proton-hyperpolarized gas media that allows rapid gas delivery in the lungs to minimize depolarization losses. The depolarization losses can be potentially further mitigated using long-lived HP states at low magnetic fields^54,55^ and using larger molecules such as diethyl ether and butane.^37,55,56^

It should also be noted that there is a reduced background signal from thermally polarized inflated lung tissue (images acquired after the inflation and depolarization of the HP state). This observation clearly shows that the HP signal overshadows the background signal, thus making the background subtraction method more robust. We anticipate that the background signal will be stronger from the thermally polarized surrounding tissues *in vivo*, and the image subtraction would work adequately.

In a separate experiment, the imaging matrix was decreased to 64 × 64 resulting in an increased pixel size of 1.6 × 1.6 mm^2^, using the same FOV of 100 × 100 mm^2^ and the same slice thickness of 50 mm. This decrease of the imaging matrix size accelerated image acquisition to sub-second speed (0.94 s scan time), while yielding similar values of the mean SNR, Fig. S4† (one should not directly compare the SNR values per pixel size of images recorded with 64 × 64- and 96 × 96-imaging matrices as multiple parameters are changed at the same time including the signal acquisition time, voxel size and the effect of HP state depolarization). These results are important as they demonstrate the feasibility of performing a ventilation MRI scan with sub-second temporal resolution. Moreover, faster scan speed is also important to minimize HP propane gas polarization losses due to fast *T*_1_ (*ca.* 0.8 s) and *T*_S_ (*ca.* 1–2 s, which have been shown to exist in HP propane gas at sufficiently low magnetic fields) at a clinically relevant pressure of 1 atm. Furthermore, the fast scan speed is also welcome from the future clinical perspective of performing the ventilation imaging scan on a single patient breath hold in a manner similar to that of the HP ^129^Xe gas contrast agent.^16^ Indeed, we have previously demonstrated the feasibility of recording 8 slices in 0.4 s on a HP propane gas phantom using a clinical 3 T GE Healthcare scanner^36^ that has a substantially faster scan speed compared to the 0.35 T scanner employed for this study, demonstrating that comprehensive multi-slice 2D scanning of the lungs may be potentially feasible in less than 1 s. Sub-second ventilation imaging was also reproducible (Fig. S7†).

**Fig. 3:**
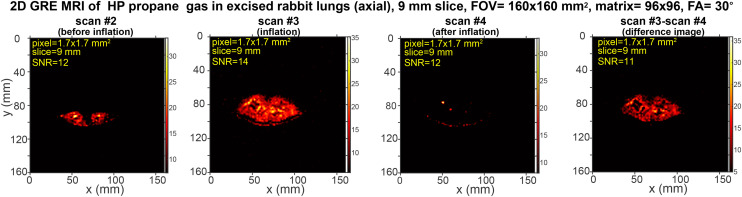
Rapid slice-selective 2D GRE images of HP propane gas (axial projection) rapidly expanding in excised rabbit lungs, acquired by utilizing a 0.35 T MRI scanner and a knee RF coil. Scan #2 was recorded before HP gas inflation, scan #3 was recorded during the inflation, and scan #4 was recorded after the HP gas inflation at 1.94 s temporal resolution. The difference image is obtained as the difference between scan #3 and scan #4. The corresponding mean SNR (see the ESI† for details) is provided. The scans were acquired with a 160 × 160 mm^2^ FOV, a 9 mm slice thickness, a 30° RF excitation pulse, a 96 × 96 imaging matrix, and post-processing interpolation to 768 × 768 pixels. A total of 16 repeat scans were recorded in 31 s, with a repetition time (TR) of 20.27 ms and an echo time (TE) of 9.75 ms. Fig. S24† shows the screenshot of the MRI scans as visualized with the 0.35 T scanner without any additional data processing.

In a separate experiment shown in Fig. 3, the slice thickness was decreased to 9 mm *versus* 50 mm (Fig. 2), while the FOV was increased to 160 × 160 mm^2^, resulting in a pixel size of 1.7 × 1.7 mm^2^. The reduced slice thickness allows scanning a section of the lung (9 mm thick) *versus* effectively recording a projection scan with a 50 mm slice thickness. The results shown with 9 mm slice thickness are important as they demonstrate the feasibility of recording MRI scans with pixel size on a mm-scale *versus* effective 2D projection image scans.

As expected, further decrease of the spatial resolution by increasing the pixel size to 2 × 2 mm^2^ (50 mm slice thickness) *via* expanding the FOV to 128 × 128 mm^2^ and maintaining an imaging matrix size of 64 × 64 yielded sub-second MRI scans with a substantially improved mean SNR up to 28 in the axial projection (in the background subtraction images, Fig. S10†), which was also reproducible (with a corresponding mean SNR of 28, Fig. S13†). Further increase of the FOV and the pixel size to 160 × 160 mm^2^ and 2.5 × 2.5 mm^2^, respectively (while maintaining other parameters the same, *i.e.*, matrix size of 64 × 64 and (50 mm slice thickness)), resulted in additional gains in the mean SNR of up to 43 (axial projection difference image in Fig. S16†). These results highlight the possibility of recording ventilation MRI scans with a high SNR over a large FOV, which is important in the context of future clinical translation of this MRI modality. Moreover, the results reported in Fig. S16a,† showing the image of the inflated lungs with a higher SNR (albeit at the expense of spatial resolution), clearly exhibited the following unexpected feature: the image recorded at inflation (SNR of 54) is followed by the image with an SNR of 26, *i.e.*, clearly showing substantial residual magnetization left after the completion of the first MRI scan that was recorded after the gas injection was stopped (note the same overall shape of the lungs in two images, further supporting the fact that the HP gas was stopped, when they were recorded). The substantial amount of retained SNR is rather unexpected because a 30° RF excitation pulse is anticipated to depolarize the non-replenishable HP state by 10-fold in 16 excitation pulses (note that 64 RF pulses were applied in total to record that image); thus, one anticipates recording only one “good-quality” image with virtually no magnetization left after the completion of 64 slice-selective RF excitation pulses employed (Fig. S16a†). We rationalize this finding by the fact that HP H_A_ and H_B_ sites are in the intermediate spin–spin coupling regime (*J*_H_A_–H_B__ is approximately equal to the chemical shift difference between H_A_ and H_B_ sites). As a result, long-lived spin states (LLSS) may be “partially” present at this field for HP propane gas produced *via* PHIP. We speculate that “partial” LLSS are mostly immune to the RF excitation pulses and may “effectively” replenish NMR-visible magnetization throughout acquisition MRI scans (and application of successive 64 RF excitation pulses), thus, resulting in the substantially lower depolarizing effect of the RF excitation pulses: however, detailed relaxation dynamics studies are certainly required to support this hypothesis.

**Fig. 4:**
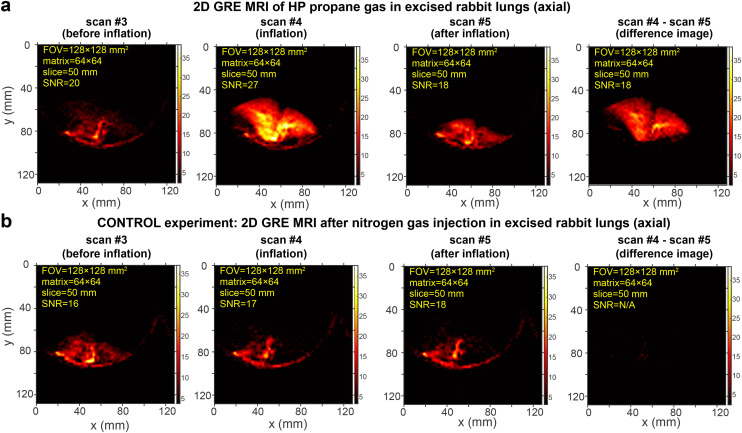
Rapid slice-selective 2D GRE images of HP propane gas rapidly expanding in excised rabbit lungs and the control study performed using non-hyperpolarized inert nitrogen gas, acquired by utilizing a 0.35 T MRI scanner and a knee RF coil. (a) Axial projection of the excised rabbit lungs recorded before inflation (scan #3), during inflation (scan #4), and after inflation (scan #5) with HP propane gas at a 1.1 s temporal resolution. (b) Corresponding control experiment, where HP propane gas was replaced with inert nitrogen gas that performs similar inflation, but carries no HP state in contrast to images shown in display a. The difference images for both HP propane and nitrogen gas experiments were obtained as the difference between scan #4 and scan #5. The corresponding mean SNR values associated with each image are reported. Note that the difference image for nitrogen gas inflation (control experiment) yields virtually no signal (as expected), and therefore, the SNR value is marked as N/A. The axial scans were acquired with a 128 × 128 mm^2^ FOV, a 50 mm slice thickness, a 30° RF excitation pulse, a 64 × 64 imaging matrix, and post-processing interpolation to 768 × 768 pixels. A total of 16 repeat scans were recorded in 18 s, with TR = 17.19 ms and TE = 8.21 ms. Fig. S25 and S26† show the screenshots of the corresponding MRI scan series (shown in displays a and b, respectively) as visualized with the 0.35 T scanner without any additional data processing.

Additional control experiments were performed with non-hyperpolarized non-hydrocarbon gas (Fig. 4). In these control experiments, we first recorded a ventilation scan with HP propane over a 128 × 128 mm^2^ FOV and an imaging matrix size of 64 × 64: a clear visualization of the lungs is seen in the difference image in Fig. 4a, while no substantial signals are seen in the difference image in the corresponding data set when HP propane gas was replaced with N_2_ gas (Fig. 4b). These results are important as they clearly show that the ventilation image is obtained truly from HP propane inflating the lungs rather than from other MRI artifacts due to rapid lung expansion.

## Future outlook

We envision that the obtained results could be additionally optimized in the future as follows. More SNR-efficient scanning approaches could be deployed (for example, compressed and parallel sensing, spiral k-space sampling, and echo-planar imaging^36^) to increase the sensitivity and speed, potentially enabling multi-slice scanning in less than 1 s.^36,57^ Furthermore, additional efforts could certainly be made to utilize water background suppression using already-established clinical protocols.^58,59^ It should also be made clear that future *in vivo* studies will likely reveal a substantial thermally polarized background signal from the lung tissue and the surrounding tissues additionally compounded by the motion artifacts that are commonly present in lung imaging.^57^ A number of already established approaches for background subtraction (for example, those employed in Dynamic Contrast Enhancement (DCE) MRI^60^) and advanced motion correction techniques^57^ have been developed to address these challenges *in vivo*. The required high scanning speed is a clear limitation of the proton-HP gas technology, and some MRI scanners may not be sufficiently fast, limiting the reach of this technology.

For future *in vivo* experiments, we envision the delivery of the gas *via* a mouthpiece with an overpressure exit path to ensure that the arriving gas can be either readily inhaled or safely exited without over-pressurization of the mouth, airways, and lungs. Noteworthily, portable handheld oxygen aluminum cans (*e.g.*, Boost Oxygen^TM^) employ such a mouthpiece and an oxygen pressure of 15 bar inside the can: no special training is required to operate such a device, which is sold without prescription in the US and other developed countries. We also envision that the exiting gas may be potentially scavenged by a carbon filter (active ongoing studies in our partnering laboratories). The can/tank that stores the mixture of unsaturated precursor and p-H_2_ can be potentially pressurized to 10 bar or lower pressure as long as a sufficiently high flow of the HP gas stream is provided for HP propane gas inhalation: the key requirement is the pressure drop to atmospheric pressure at the mouthpiece for safe operation of such a device. We also envision that the can/tank, containing the mixture of the unsaturated precursor and p-H_2_ can be either placed inside the bore of the MRI scanner (using all-non-magnetic components and single-dose capacity) or placed in the equipment room of the MRI scanner (for a multi-dose capacity). It is also envisioned that HP propane gas can be potentially administered as a single bolus of pure HP gas or as a dilute mixture of HP propane gas with medical air for multi-inhalation protocols. Both approaches have their own merits. Noteworthily, a substantially more dense HP ^129^Xe gas is well tolerated in a single-dose inhalation of up to 1 liter,^61,62^ indicating that less dense propane can be also potentially similarly well tolerated. We anticipate no substantial difference between any change in lifetime of HP propane gas caused by dilution with oxygen, because dilution-induced changes in gas viscosity and molecular properties are anticipated to be negligible at physiologically relevant pressures and temperatures.^33,55,63,64^

Despite the fact that the results reported here were obtained with a *P*_H_ of only 0.5–1%, while clinical studies with HP ^129^Xe typically utilize 20–30% polarized ^129^Xe, high sensitivity of proton-hyperpolarized scanning becomes possible due to the higher gyromagnetic ratio and the greater natural abundance of protons compared to those of ^129^Xe: specifically, we anticipate that two HP propane gas protons will yield more than 10 times more SNR compared to HP ^129^Xe gas at the same nominal isotopic concentration and polarization.^58,59^ The additional gains in *P*_H_ are also potentially feasible through improved efficiency of pairwise p-H_2_ addition to propylene *via* a more advanced catalyst design.^65^ HP propane gas could be quickly produced on demand at a low cost,^36^ and it can be readily deployed on clinical MRI scanners without any modifications (including the low-field 0.35 T MRI scanner employed here), highlighting key advantages of this technology. On the other hand, the fast depolarization of the propane HP state demands establishing the HP agent production in close proximity to the MRI scanner, which is a clear disadvantage compared to HP ^129^Xe production, which can be performed far away from the imaging suite. Moreover, HP ^129^Xe exhibits distinct *in vivo* chemical shifts (gas, tissue membrane, and red-blood cell phases, and potentially more using the biosensor approach^66^), whereas propane protons are expected to have no substantial chemical shift differences in those environments, thus, making measurements of gas perfusion in lungs^16,67^ using HP propane hardly possible.

We have employed 99% pure propylene and 99.999% hydrogen for these experiments, which we believe meet the purity requirements for GMP production of propane gas (typically 99%). The heterogeneous catalyst allows producing a catalyst-free stream of HP propane gas, although rigorous excipient testing of the key components (clean food-grade copper tubing, food-grade titania, rhodium metal, and brass connectors) employed in the hyperpolarizer fluid path design is required for future clinical translation. Propane gas is already regulated by the FDA and many other regulatory bodies (designated E944 food additive label in Europe), and propane is generally recognized as safe (GRAS).^68^ Propane gas is approved for unlimited use in food applications as a propellant gas and as an ingredient under GMP production. Propane gas is a non-toxic asphyxiant. Indeed, a randomized ninety-day inhalation toxicity study at 10 000 ppm gas-phase concentration revealed no observable systemic effects.^69^ Propane gas was also safely administered in a concentration up to 20% for one minute.^70,71^ While these previous studies serve as a good foundation that hyperpolarized propane gas can likely be safely administered for MRI scanning purposes, we note that there is currently no regulatory approval for such examination. We envision that the possible side effects of HP propane gas administration certainly need to be studied prior to clinical utilization of this potential inhalable contrast agent for functional lung imaging utilization. Moreover, careful MRI suite planning with respect to the dosing, administration, flammability, and scavenging using a carbon filter must be addressed to maintain the safety of the personnel and patients near equipment that is known to generate kV potentials and arcing.^72^

## Conclusions

In summary, the feasibility of high-resolution ventilation proton MRI of HP propane in excised rabbit lungs has been demonstrated with high resolution (1 × 1 × 50 mm^3^ and 1.7 × 1.7 × 1 × 9 mm^3^ voxel size) and ultrafast scan speed (1.7–1.9 s) using a clinical 0.35 T MRI scanner without any specialized hardware or pulse sequences. Moreover, the feasibility of sub-second scan speed has been demonstrated, albeit at a slightly reduced spatial resolution of a 1.6 × 1.6 × 50 mm^3^ voxel size. These results bode well for future biomedical translation of HP propane gas and other proton-hyperpolarized inhalable contrast media.^37^

## Ethical statement

All animal procedures were performed in accordance with the Guidelines for Care and Use of Laboratory Animals of “Wayne State University” and approved by the Animal Ethics Committee of “IACUC (23-01-5364)

## Author contributions

Md Raduanul H. Chowdhury: software, data curation, formal analysis, visualization, and writing – original draft writing, review & editing; Clementinah Oladun: software, data curation, formal analysis, and visualization; Nuwandi M. Ariyasingha: software, investigation, data curation, formal analysis, and visualization; Anna Samoilenko: investigation; Tarek Bawardi: data curation, formal analysis, visualization, and writing – original draft writing, review & editing; Dudari B. Burueva: review & editing; Oleg G. Salnikov: review & editing and methodology; Larisa M. Kovtunova: review & editing and methodology; Valerii I. Bukhtiyarov: review & editing; Zhongjie Shi: investigation and methodology; Kehuan Luo: investigation and methodology; Sidhartha Tan: project administration, resources, supervision, and review & editing; Juri G. Gelovani: review & editing and funding acquisition; Igor V. Koptyug: supervision and review & editing; Boyd M. Goodson: project administration, funding acquisition, resources, and review & editing; Eduard Y. Chekmenev: conceptualization, data curation, formal analysis, funding acquisition, investigation, methodology, project administration, resources, software, supervision, validation, writing, and review & editing.

## Data availability

All raw DICOM MRI scans that are presented in the main text and the ESI† can be found at https://doi.org/10.17632/dbkf9pfvkh.1.

## Conflicts of interest

BMG and EYC declare stake ownership in XeUS Technologies, Ltd. EYC declares a stake of ownership in Vizma Life Sciences.

## Supplementary Material

AN-149-D4AN01029A-s001
