# Supplementary material for: Rapid lung ventilation MRI using parahydrogen-induced polarization of propane gas
Source: Analyst. 2024 Nov 12;149(24):5832–42. doi: 10.1039/d4an01029a (PMC11563306; doi:10.1039/d4an01029a)
Supplement: AN-149-D4AN01029A-s001 [file AN-149-D4AN01029A-s001.pdf]

Supplementary Information (SI) for Analyst.
This journal is © The Royal Society of Chemistry 2024

Supporting Information For

Rapid Lung Ventilation MRI Using Parahydrogen-Induced Polarization of Propane Gas

Md Raduanul H. Chowdhury,[a] Clementinah Oladun,[a] Nuwandi M. Ariyasingha,[a] Anna Samoilenko,[a] Tarek Bawardi,[a] Dudari B. Burueva,[b] Oleg G. Salnikov,[b] Larisa M. Kovtunova,[b,c] Valerii I. Bukhtiyarov,[c] Zhongjie Shi,[d] Kehuan Luo,[d] Sidhartha Tan,[d] Juri G. Gelovani,[a,e] Igor V. Koptyug,[b] Boyd M. Goodson,[f] Eduard Y. Chekmenev*[a]

[a.] *Department of Chemistry, Integrative Biosciences (Ibio), Wayne State University, Karmanos Cancer Institute (KCI), Detroit, Michigan 48202, United States*
E-mails: chekmenevlab@gmail.com
[b.] *International Tomography Center, SB RAS, 3A Institutskaya St., Novosibirsk 630090, Russia*
[c.] *Boreskov Institute of Catalysis SB RAS, 5 Acad. Lavrentiev Pr., Novosibirsk 630090, Russia*
[d.] *Department of Pediatrics, Wayne State University, Detroit, Michigan 48202, United States*
[e.] *United Arab Emirates University, Al Ain, United Arab Emirates*
[f.] *School of Chemical & Biomolecular Sciences, Materials Technology Center, Southern Illinois University, Carbondale, IL 62901, USA, United States*

Table of Contents

1. Mean signal-to-noise-ratio (SNR) calculation procedure S3

2. Additional rabbit lungs images obtained using HP propane gas and different imaging parameters S4

2a. Reproducibility of HP propane MRI with 100×100 mm^2 FOV and 1×1 mm^2 pixel size S4

2b. HP propane MR images with 100×100 mm^2 FOV and 1.6×1.6 mm^2 pixel size S6

2c. HP propane MR images with 128×128 mm^2 FOV and 2×2 mm^2 pixel size S10

2d. HP propane MR images with 160×160 mm^2 FOV and 2.5×2.5 mm^2 pixel size S14

3. Screenshots of HP propane MR images displayed in the main text Figure 2 .... S18

4. MATLAB image processing S22

5. References Used in Electronic Supporting Information (ESI) S27

1. Mean signal-to-noise-ratio (SNR) calculation procedure

SNR is a key parameter in the evaluation of image quality and confirmation of the hyperpolarized (HP) gas presence. The SNR values were computed for all images in the figures of the main text (**Figures 2-4)** and SI (**Figures S1, S4, S7, S10, S13, S16, and S19**).

All image pixels are analyzed for their intensities. First, the region of the image (8×8 pixels in size), where no signal is seen by a user is selected to compute the RMS noise value. This RMS noise value is employed for identifying pixels that contain a signal. When a pixel intensity exceeds a threshold value (computed based on the RMS noise value), the pixel signal is added to the overall calculation of SNR (specifically, the signal is added to the total sum of all signal values from all pixels exceeded the threshold value); in case if the pixel intensity is below the threshold value, it is discarded from the SNR calculation. Next, the mean signal is computed by finding the mean values of all the pixels exceeding the threshold value. Finally, the SNR is computed by dividing the mean signal value by the value of RMS noise.

signal-to-noise ratio (SNR) = (mean *signal*/*RMS* (*noise)*) (Eq. S1)

This approach mitigates the need for the region of interest (ROI) selection by an end user.

We used an automated MATLAB code to process all the images and calculate the SNR values. The full code used for processing of the images can be found in section 4 of this ESI document.

2. Additional rabbit lungs images obtained using HP propane gas and different imaging parameters

This section contains additional excised rabbit lungs MRI scans obtained using HP propane gas. It also contains numerous reproducibility scans and the corresponding screenshots of the images processed by the MRI scanner software before any data post-processing using MATLAB.

2a. Reproducibility of HP propane MRI with 100×100 mm^2 FOV and 1×1 mm^2 pixel size

Figure S1a and **S1b** present the reproducibility results of the scan presented in **Figure 2** of the main text: the axial and coronal projection of the excised rabbit lungs MR images using HP propane. The mean SNR of **Figure S1a** is 14 in before inflation, 14 in during inflation, and 12 in after inflation images, respectively, compared to the mean SNR of 14, 16, and 12 in the corresponding images in the main text **Figure 2a**. In **Figure S1b** the mean SNR is 13 in before inflation, 12 in during inflation, and 13 in after inflation images, respectively, compared to mean SNR of 12, 15, and 13 in the corresponding images in the main text **Figure 2b**. The imaging parameters used in these experiments were: 16 slices (total 27 seconds), 96×96 imaging matrix, 100×100 mm^2 field of view (FOV), 30° slice-selective excitation RF pulse, spectral width 10.42 kHz, repetition time (TR) 17.8 ms, echo time (TE) 8.5 ms. The difference images were obtained as the differences of scan #5 and scan #6 for axial and scan #4 and scan #5 for coronal projections, respectively, as there was virtually no HP signal left in the after inflation images, which yielded mean SNR of 11 (axial) and 12 (coronal) in **Figure S1** compared to 13 (axial) and 12 (coronal) in the main text **Figures 2a** and **2b**. The 16 axial and coronal images processed with the MRI scanner software before any MATLAB data processing are shown in **Figure S2** and **Figure S3**, respectively.

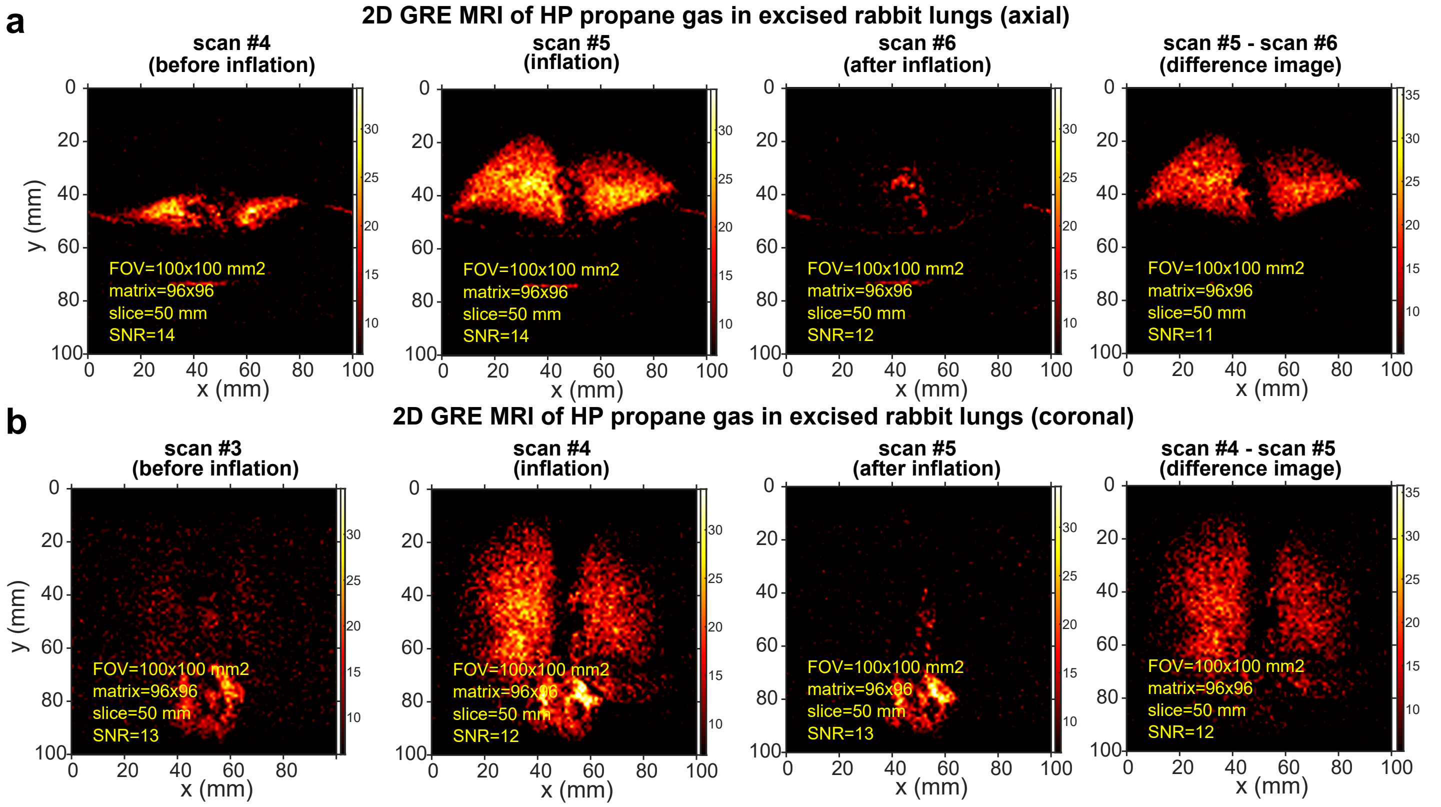

Figure S1. Ultra-fast slice-selective 2D GRE images of HP propane gas injected in excised rabbit lungs acquired by utilizing a 0.35 T MRI scanner and knee RF coil. a) Axial projection of the excised rabbit lungs recorded before inflation (scan #4), during inflation (scan #5), and after inflation (scan #6) with HP propane gas at 1.7 s temporal resolution. b) Corresponding scans #3, #4, and #5 from coronal projection. The difference image for axial projection was obtained as the difference of scan #5 and scan #6 (shown in **Figure S2**), and for coronal projection, the difference between scan #4 and scan #5 was taken (shown in **Figure S3**). The corresponding mean SNR values associated with each image are reported. All the images from both axial and coronal projections were acquired with a 100×100 mm^2 FOV, slice thickness of 50 mm, 30° slice-selective RF excitation pulse, 96×96 imaging matrix, and post-processing image interpolation to 768×768 pixels.

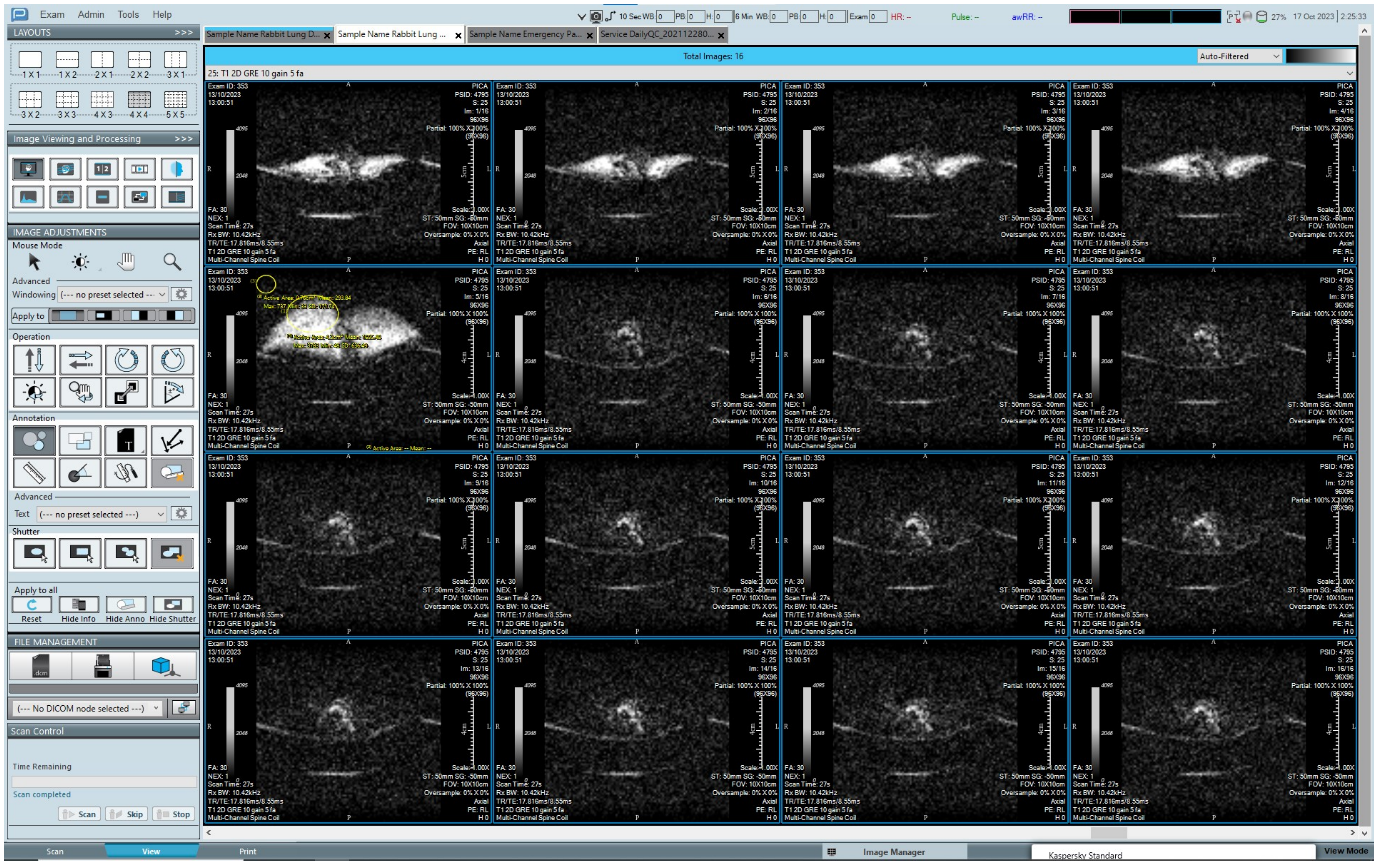

Figure S2. Screenshot of images processed by the MRI scanner software during the experiment of **Figure S1a** (axial projection, 1×1 mm^2 pixel size) without any data post-processing.

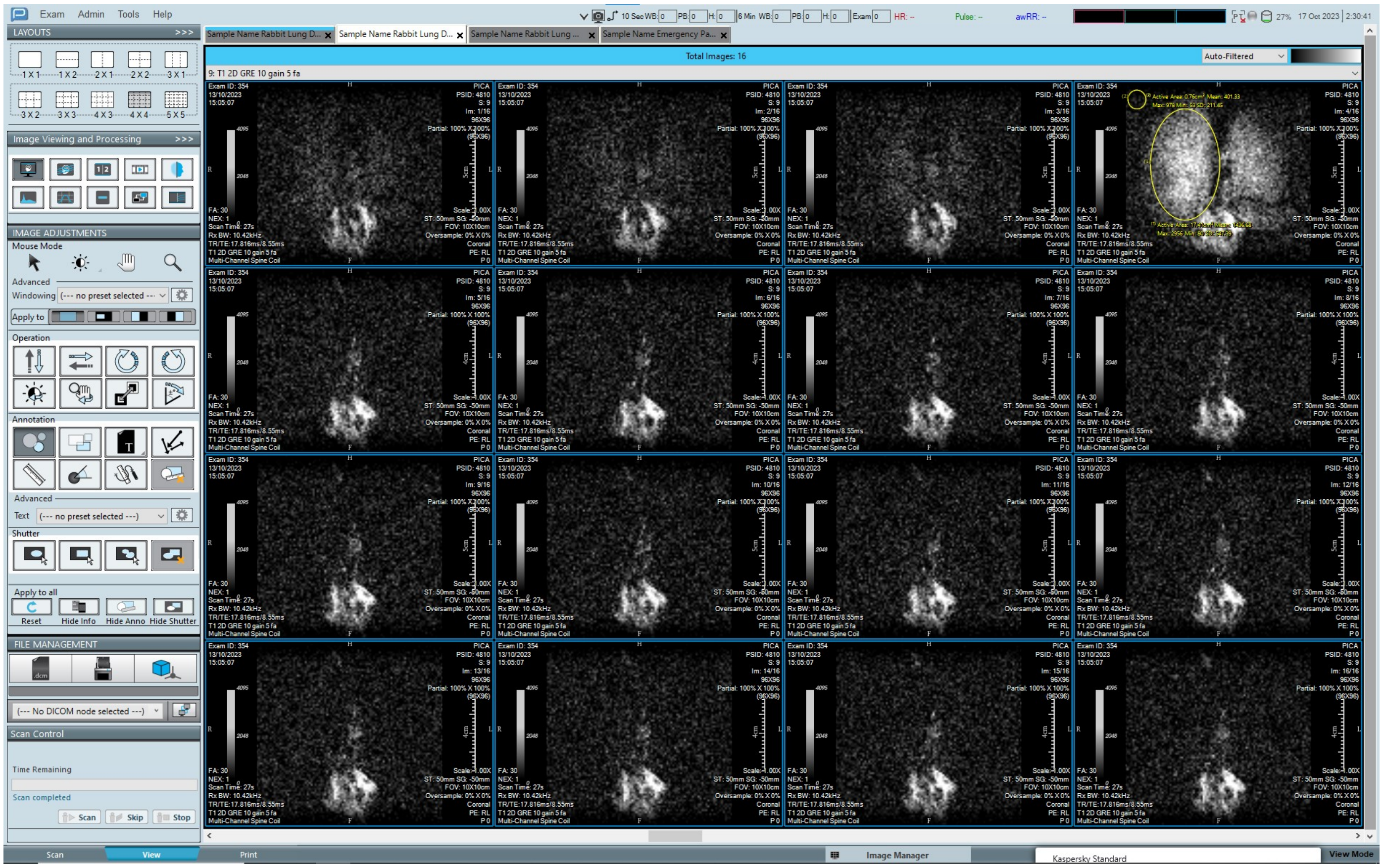

Figure S3. Screenshot of images processed by the MRI scanner software during the experiment of **Figure S1b** (coronal projection, 1×1 mm^2 pixel size) without any data post-processing.

2b. HP propane MR images with 100×100 mm^2 FOV and 1.6×1.6 mm^2 pixel size

Experiment #1

Figure S4a and **S4b** present the axial and coronal projection MR images of the excised rabbit lungs using HP propane. The mean SNR of **Figure S4a** is 17 in the image acquired before inflation, 21 in the image acquired during inflation, and 14 in image recorded after inflation images, respectively. In **Figure S4b**, the mean SNR is 12 in the image recorded before inflation, 11 in the image recorded during HP gas inflation, and 13 in the image acquired after inflation images, respectively. The imaging parameters used in these experiments were: 16 slices (total 15 seconds), 64×64 imaging matrix, 100×100 mm^2 field of view (FOV), 30° slice-selective excitation RF pulse, spectral width 10.42 kHz, repetition time (TR) 14.7 ms, echo time (TE) 6.97 ms. The difference images were obtained as the differences of scans #6 and #7 for axial, and scans #6 and #7 for coronal projections, respectively, which yielded a mean SNR of 15 (axial) and 11 (coronal) in **Figures S4a** and **S4b**. The 16 axial and coronal images processed with the MRI scanner software before any MATLAB data processing are shown in **Figure S5** and **Figure S6**, respectively.

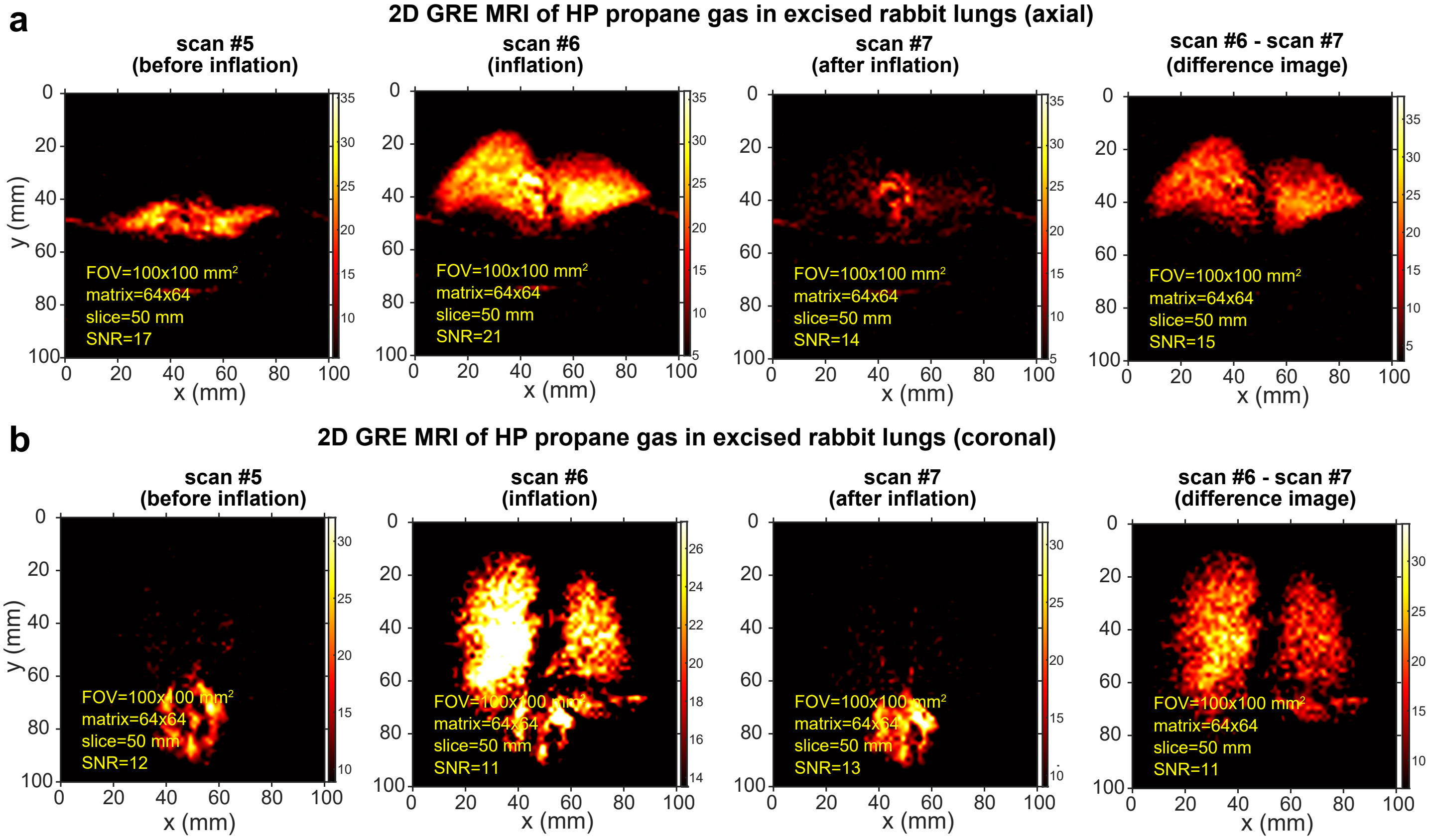

Figure S4. Sub-second slice-selective 2D GRE images of HP propane gas injected in excised rabbit lungs acquired by utilizing a 0.35 T MRI scanner and knee RF coil. a) Axial projection of the excised rabbit lungs recorded before inflation (scan #5), during inflation (scan #6), and after inflation (scan #7) with HP propane gas at 0.94 s temporal resolution. b) Corresponding scans #5–#7 from coronal projection. The difference image for axial projection was obtained as the difference of scan #6 and scan #7 (shown in **Figure S5**), and for coronal projection, the difference between scan #6 and scan #7 was taken (shown in **Figure S6**). The corresponding mean SNR values associated with each image were obtained as described above. All the images from both axial and coronal projections were acquired with a 100×100 mm^2 FOV, slice thickness of 50 mm, 30° slice-selective RF excitation pulse, 64×64 imaging matrix, and post-processing image interpolation to 768×768 pixels. The image series was acquired in 15 seconds to complete acquisition of 16 repeat scans, with a TE of 14.7 ms and a TR of 6.97 ms.

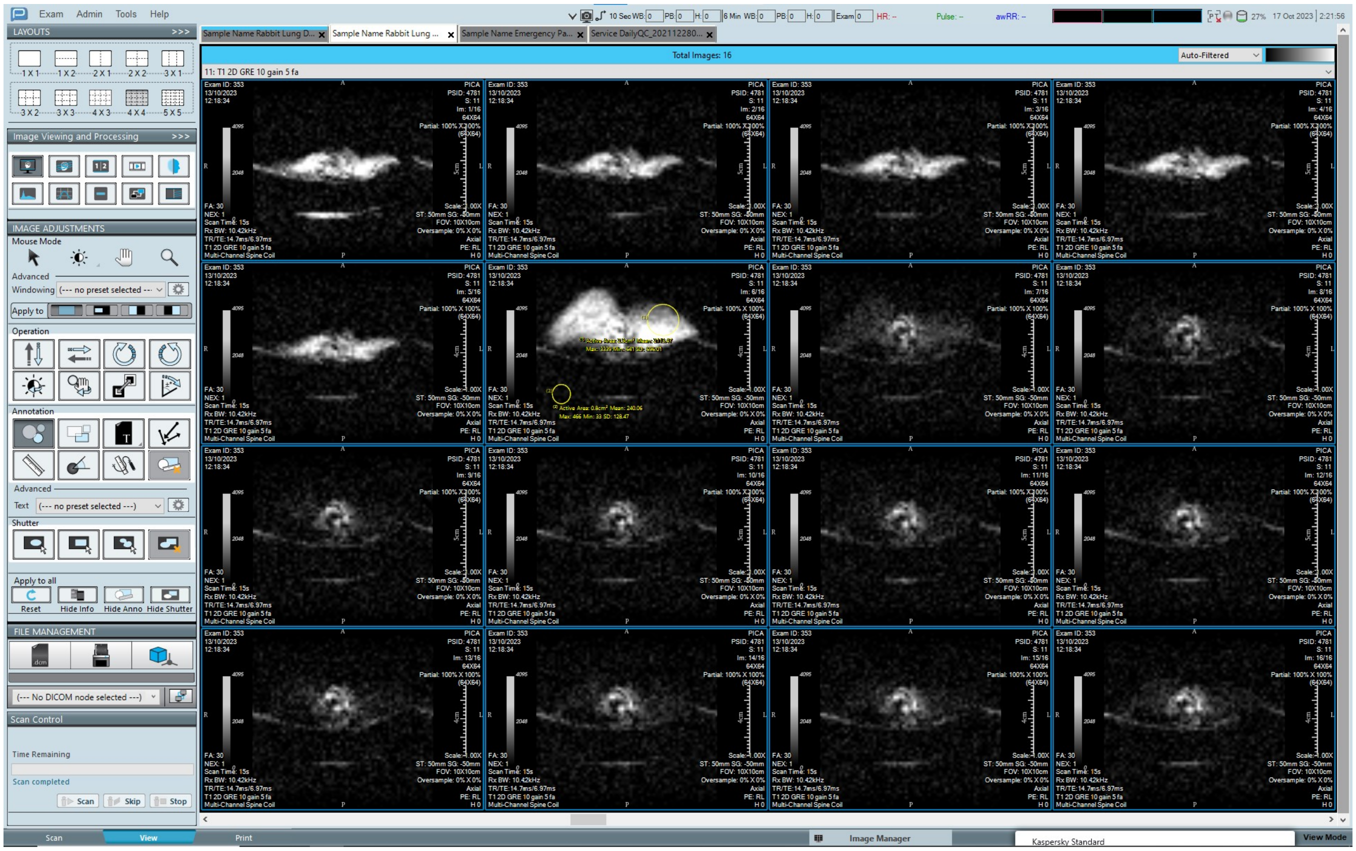

Figure S5. Screenshot of images processed by the MRI scanner software during the experiment of **Figure S4a** (axial projection, 1.6×1.6 mm^2 pixel size) without any data post-processing.

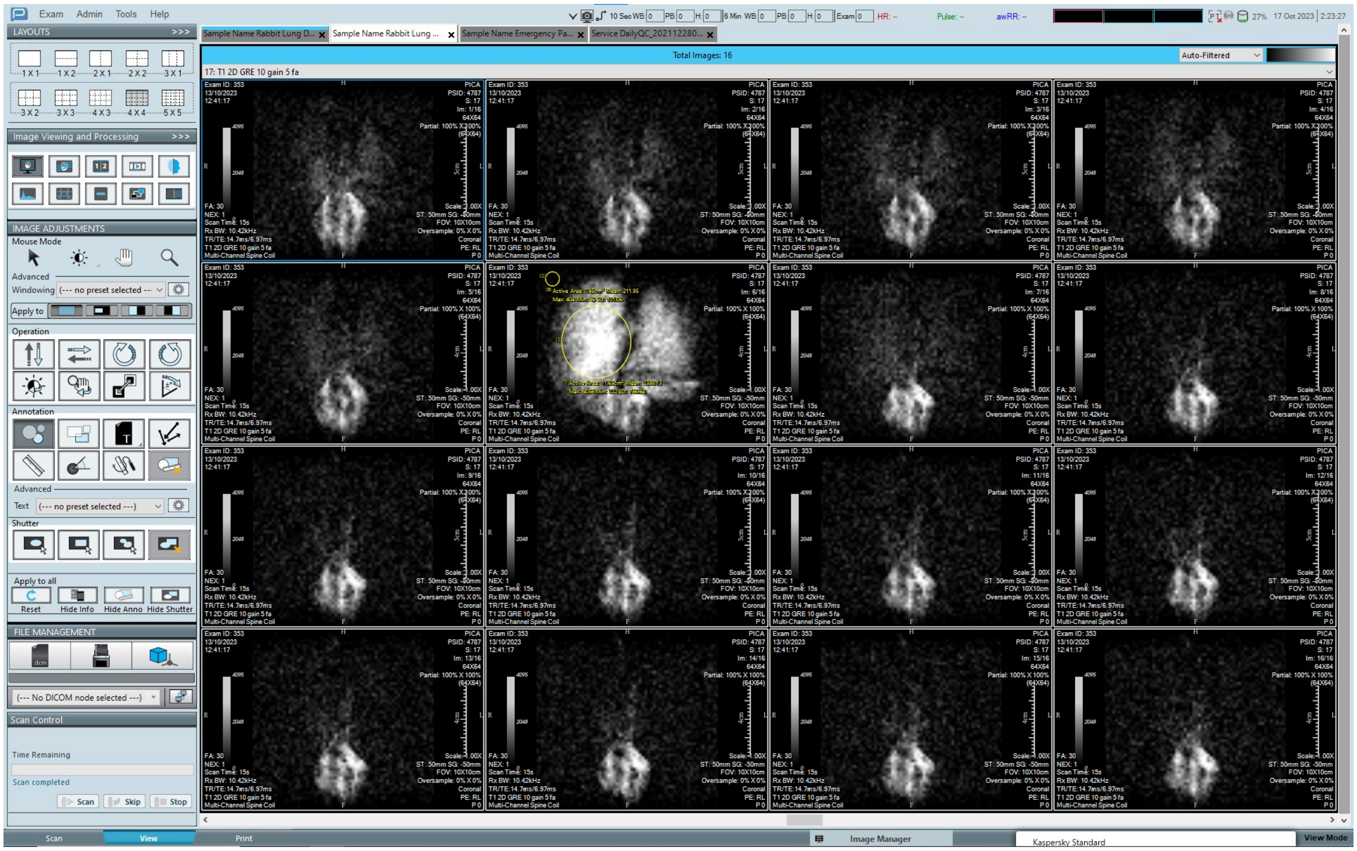

Figure S6. Screenshot of images processed by the MRI scanner software during the experiment of **Figure S4b** (coronal projection, 1.6×1.6 mm^2 pixel size) without any data post-processing.

Experiment #2

Figure S7a and **S7b** present the axial and coronal projection MR images of the excised rabbit lungs using HP propane. The mean SNR of images in **Figure S7a** was as follows: 15 in the image obtained before inflation, 20 in the image obtained during inflation, and 12 in the image obtained after inflation images, respectively, compared to **Figure S4a**, exhibiting a mean SNR of 17, 21, and 14 in the corresponding images, clearly highlighting good back-to-back scan reproducibility. In **Figure S7b**, the mean SNR is 14 in the image recorded before inflation, 11 in the image recorded during inflation, and 14 in the image recorded after inflation images, respectively, compared to 12, 11, and 13 in the corresponding images in **Figure S4b**. The imaging parameters used in these experiments were: 16 slices (total 15 seconds), 64×64 imaging matrix, 100×100 mm^2 field of view (FOV), 30° slice-selective excitation RF pulse, spectral width 10.42 kHz, repetition time (TR) 14.7 ms, echo time (TE) 6.97 ms. The difference images were obtained as the differences of scan #6 and scan #8 for axial (as there was residual signal in the after inflation axial image #7) and scan #6 and scan #7 for coronal projections, respectively, which yielded mean SNR of 14 (axial) and below 10 (labeled as N/A, in coronal) in **Figures S7a** and **S7b** compared to 15 (axial) and 11 (coronal) in **Figures S4a** and **S4b**. The 16 axial and coronal images processed with the MRI scanner software before any MATLAB data processing are shown in **Figure S8** and **Figure S9**, respectively.

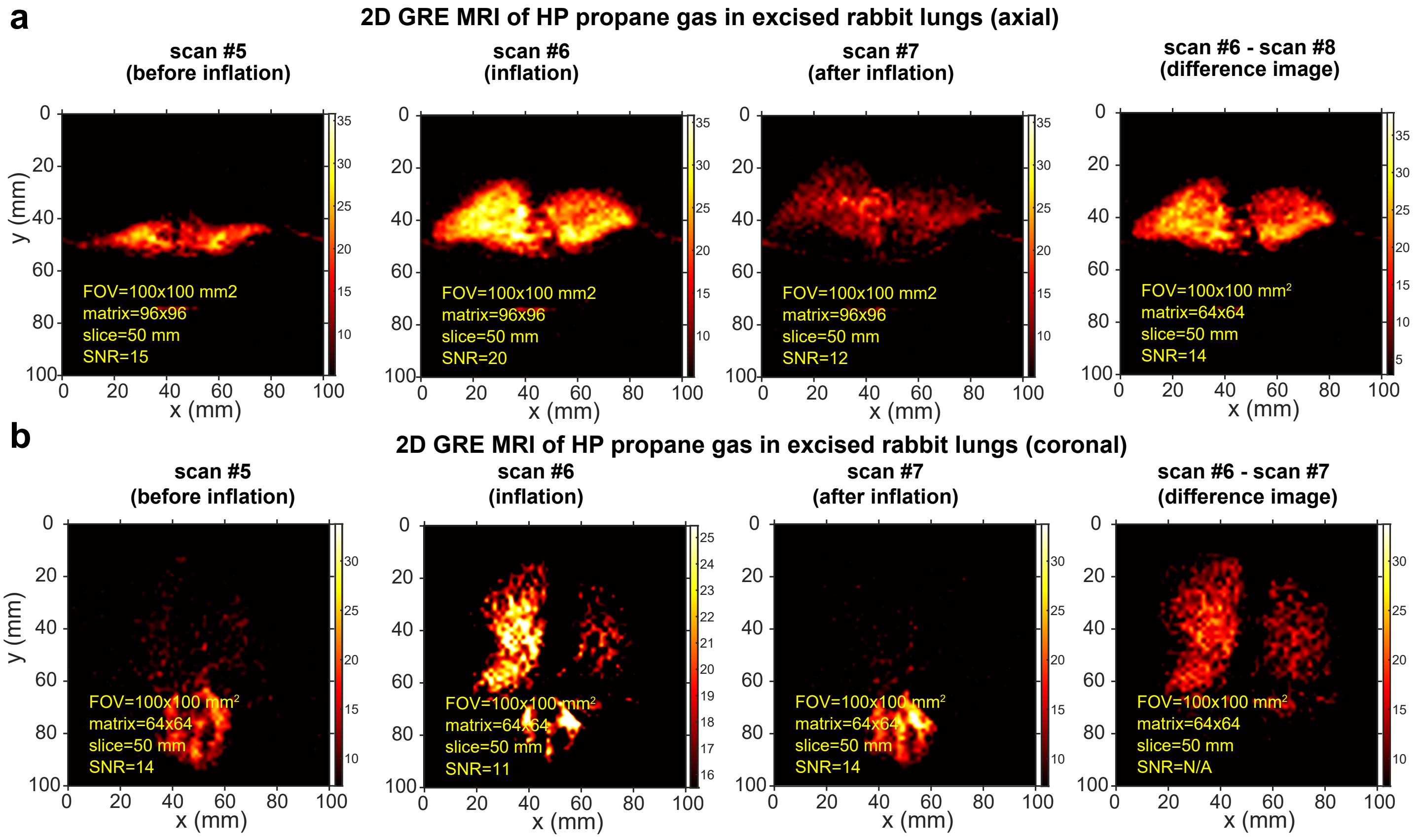

Figure S7. Sub-second slice-selective 2D GRE images of HP propane gas injected in excised rabbit lungs acquired by utilizing a 0.35 T MRI scanner and knee RF coil. a) Axial projection of the excised rabbit lungs recorded before inflation (scan #5), during inflation (scan #6), and after inflation (scan #7) with HP propane gas at 0.94 s temporal resolution. b) Corresponding scans from coronal projection. The difference image for axial projection was obtained as the difference of scan #6 and scan #7. For coronal projection difference image, the difference between scan #6 and scan #7 was taken (shown in **Figure S9**). All the images from both axial and coronal projections were acquired with a 100×100 mm^2 FOV, slice thickness of 50 mm, 30° slice-selective RF excitation pulse, 64×64 imaging matrix, and post-processing image interpolation to 768×768 pixels. The image series was acquired in 15 seconds to complete acquisition of 16 repeat scans, with a TE of 14.7 ms and a TR of 6.97 ms.

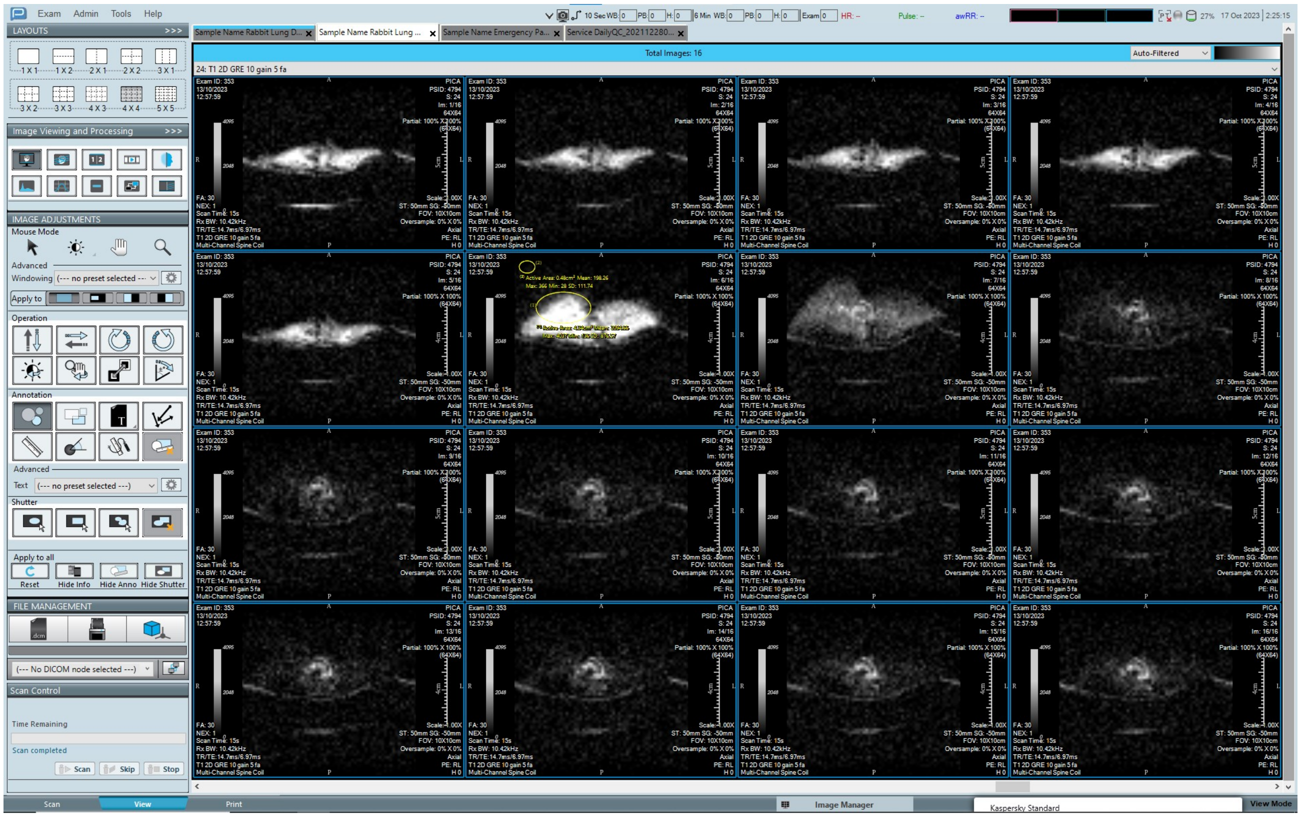

Figure S8. Screenshot of images processed by the MRI scanner software during the experiment of **Figure S7a** (axial projection, 1.6×1.6 mm^2 pixel size) without any data post-processing.

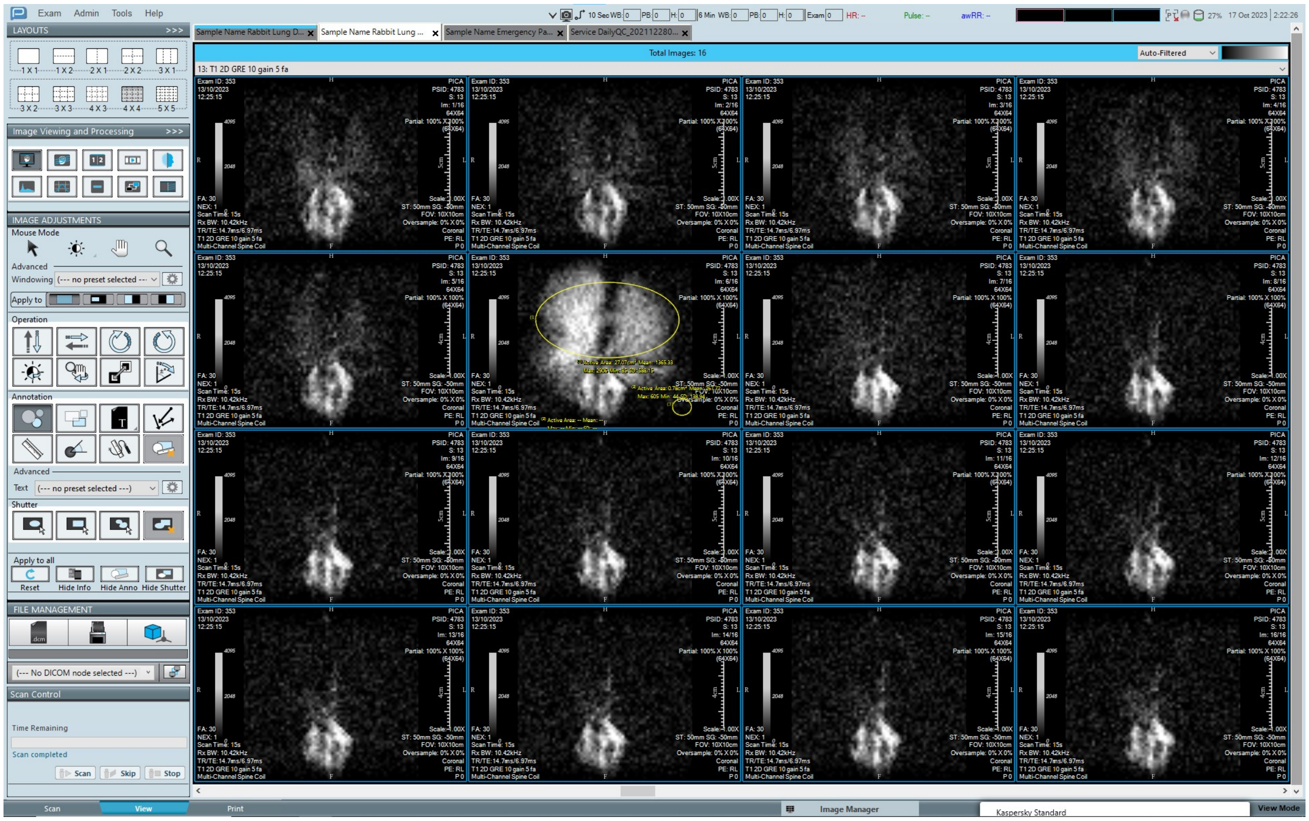

Figure S9. Screenshot of images processed by the MRI scanner software during the experiment of **Figure S7b** (coronal projection, 1.6×1.6 mm^2 pixel size) without any data post-processing.

2c. HP propane MR images with 128×128 mm^2 FOV and 2×2 mm^2 pixel size

Experiment #1

Figure S10a and **S10b** present the axial and coronal projection MRI images of the excised rabbit lungs using HP propane gas. The mean SNR of the images shown in **Figure S10a** was 25 in before inflation, 39 in during inflation, and 16 in after inflation images, respectively. In **Figure S10b**, the mean SNR is 14 in before inflation, 16 in during inflation, and 18 in after inflation images, respectively. The imaging parameters used in these experiments were: 16 slices (total 15 seconds), 64×64 imaging matrix, 128×128 mm^2 field of view (FOV), 30° slice-selective RF excitation pulse, spectral width 10.42 kHz, repetition time (TR) 14.7 ms, echo time (TE) 6.97 ms. The difference images were obtained as the differences of scan #7 and scan #9 for axial (as there was residual signal in the after inflation axial image #8) and scan #7 and scan #9 for coronal projections, respectively, which yielded mean SNR of 28 (axial) and 11 (coronal) in **Figure S10**. The 16 axial and coronal images processed with the MRI scanner software before any MATLAB data processing are shown in **Figure S11** and **Figure S12**, respectively.

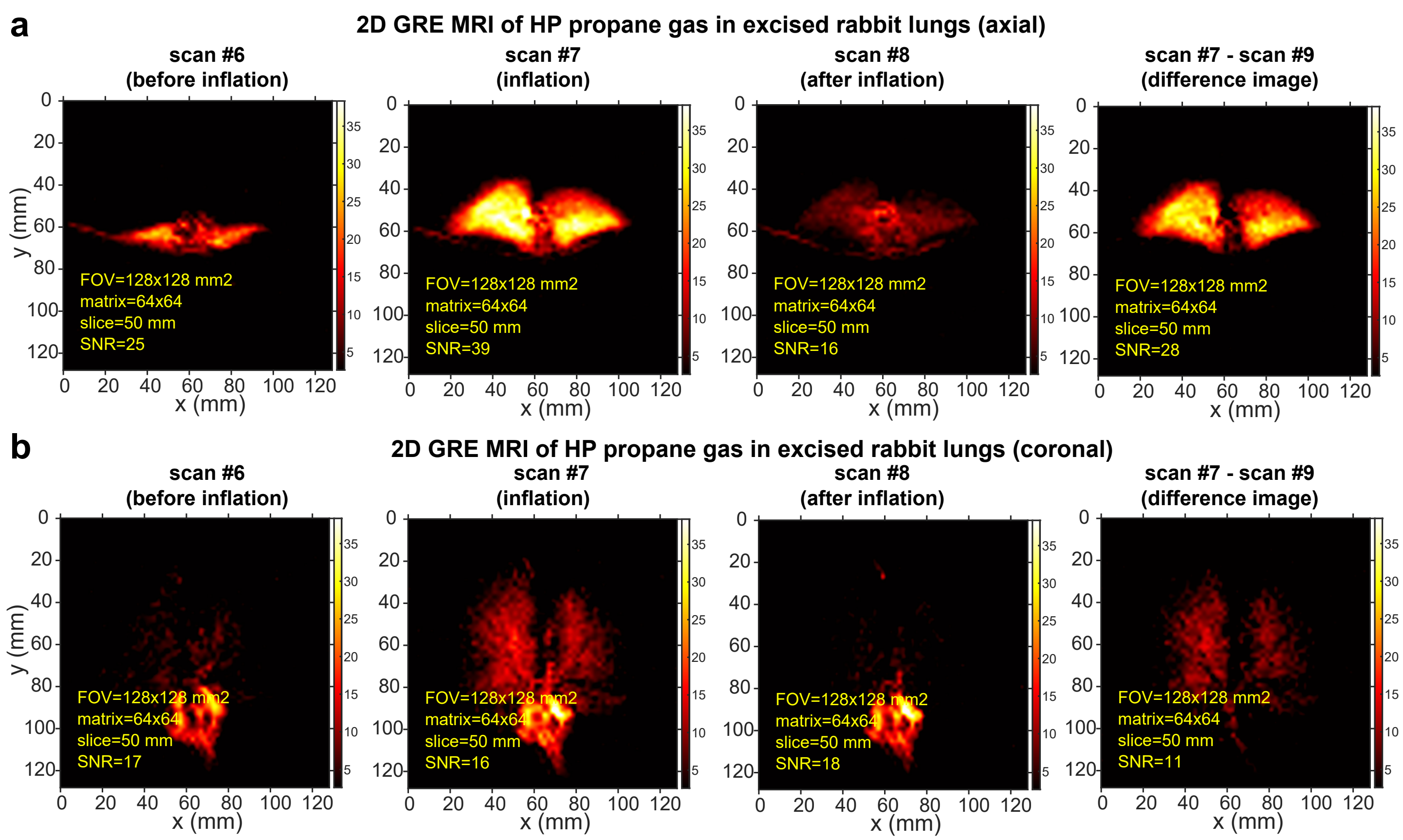

Figure S10. Sub-second slice-selective 2D GRE images of HP propane gas injected in excised rabbit lungs acquired by utilizing a 0.35 T MRI scanner and knee RF coil. a) Axial projection of the excised rabbit lungs recorded before inflation (scan #6), during inflation (scan #7), and after inflation (scan #8) with 0.94 s temporal resolution. b) Corresponding scans from coronal projection. The difference image for axial projection was obtained as the difference of scan #7 and scan #9, because of the residual HP signal presence in after inflation scan #8 (shown in **Figure S11**). For coronal projection difference image, the difference between scan #7 and scan #9 was taken (shown in **Figure S12**). All the images from both axial and coronal projections were acquired with a 128×128 mm^2 FOV, slice thickness of 50 mm, 30° slice-selective RF excitation pulse, 64×64 imaging matrix, and post-processing image interpolation to 768×768 pixels.

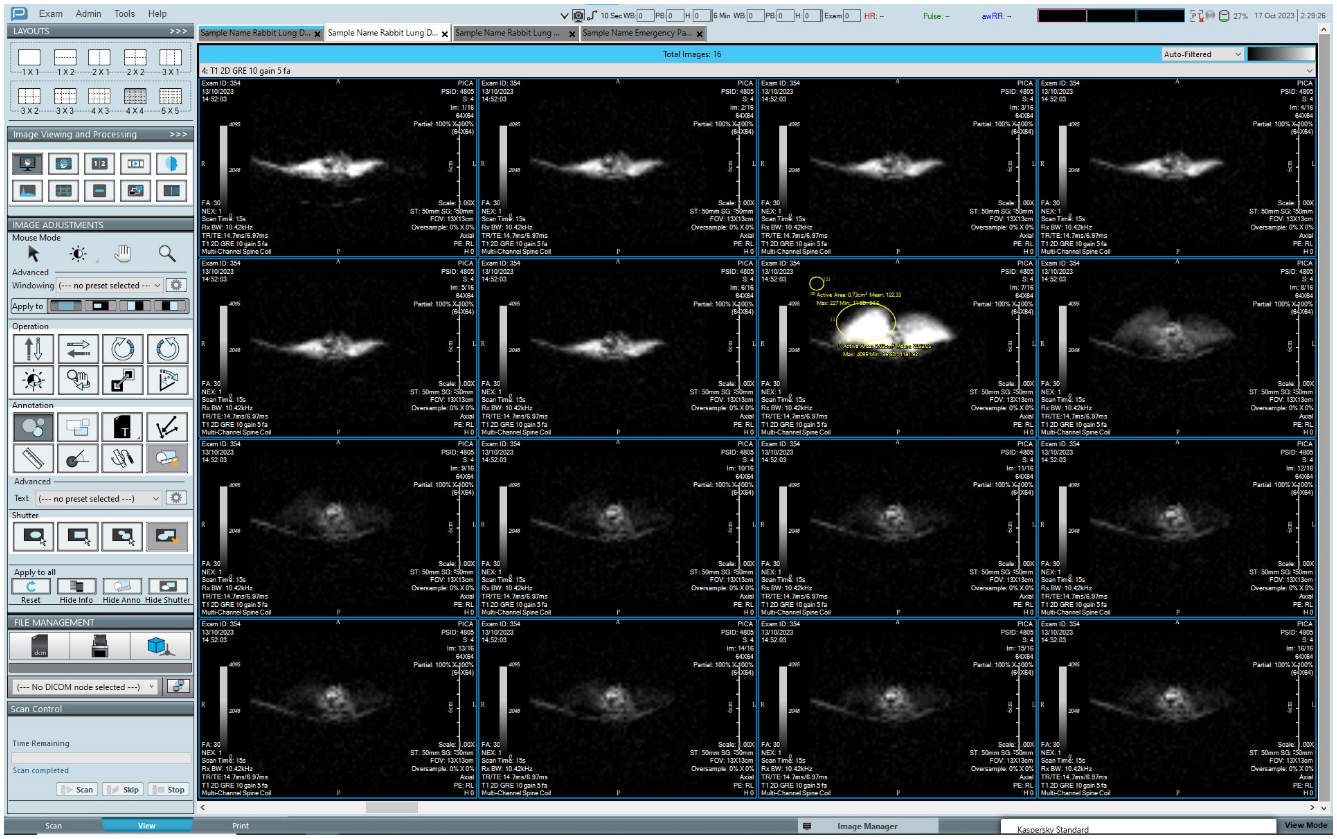

Figure S11. Screenshot of images processed by the MRI scanner software during the experiment of **Figure S10a** (axial projection, 2×2 mm^2 pixel size) without any data post-processing.

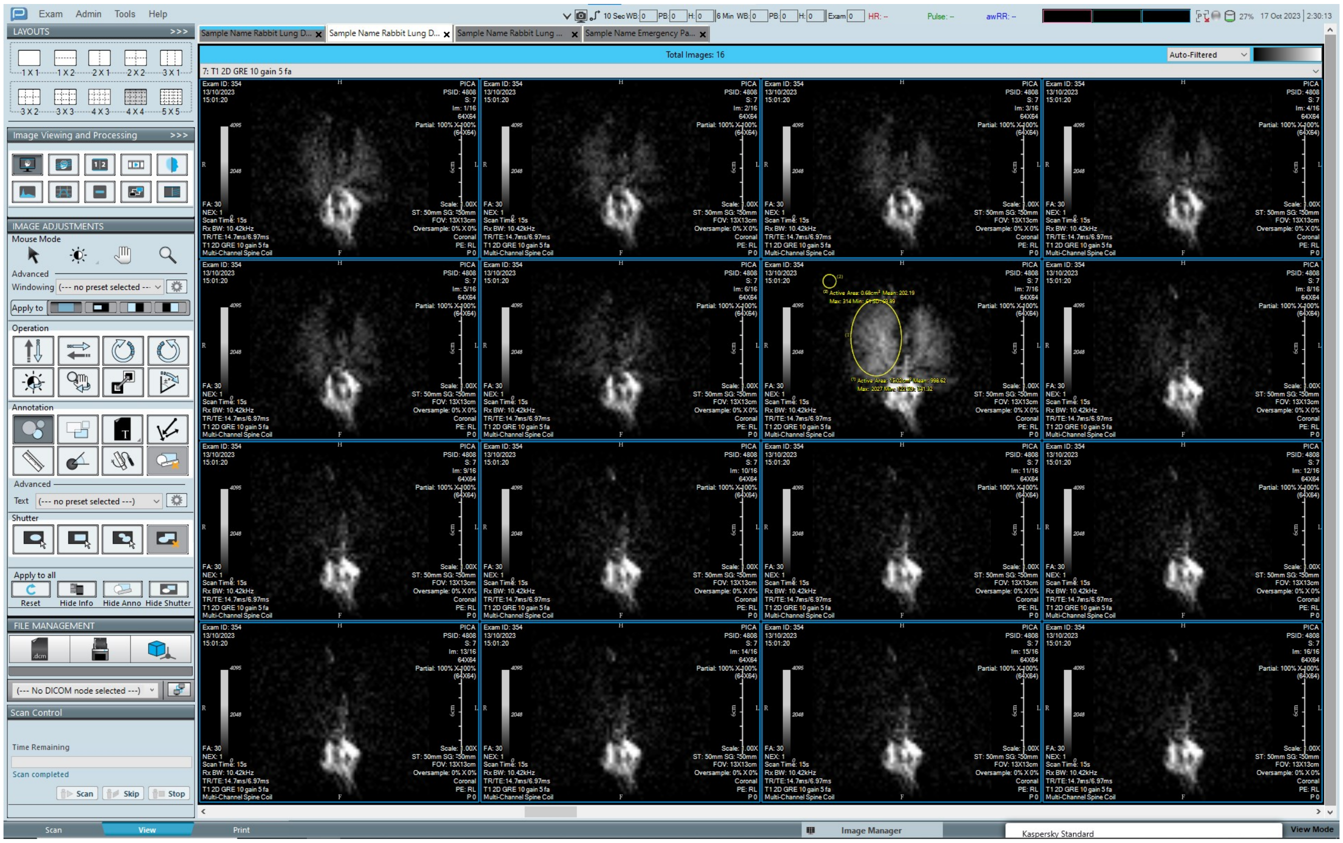

Figure S12. Screenshot of images processed by the MRI scanner software during the experiment of **Figure S10b** (coronal projection, 2×2 mm^2 pixel size) without any data post-processing.

Experiment #2

Figure S13a and **S13b** present the axial and coronal projection MR images of the excised rabbit lungs using HP propane. The mean SNR of the images shown in **Figure S13a** was 20 in before inflation, 36 in during inflation, and 16 in after inflation images, respectively, compared to **Figure S10a** mean SNR of 25, 39, and 16 in the corresponding images. In **Figure S13b**, the mean SNR is 11 in before inflation, 11 in during inflation, and below 10 (labeled as N/A) in after inflation images, respectively, compared to 17, 16, and 18 in the corresponding images in **Figure S10b**. The imaging parameters used in these experiments were: 16 slices (total 15 seconds), 64×64 imaging matrix, 128×128 mm^2 field of view (FOV), 30° slice-selective RF excitation pulse, spectral width 10.42 kHz, repetition time (TR) 14.7 ms, echo time (TE) 6.97 ms. The difference images were obtained as the differences of scan #6 and scan #8 for both axial and coronal projections (as there was residual signal in the after inflation images #7), which yielded mean SNR of 28 (axial) and 10 (coronal) in **Figure S13** compared to 28 (axial) and 11 (coronal) in **Figures S10a** and **S10b**, clearly shown good reproducibility of the back-to-back scans The 16 axial and coronal images processed with the MRI scanner software before any MATLAB data processing are shown in **Figure S14** and **Figure S15**, respectively.

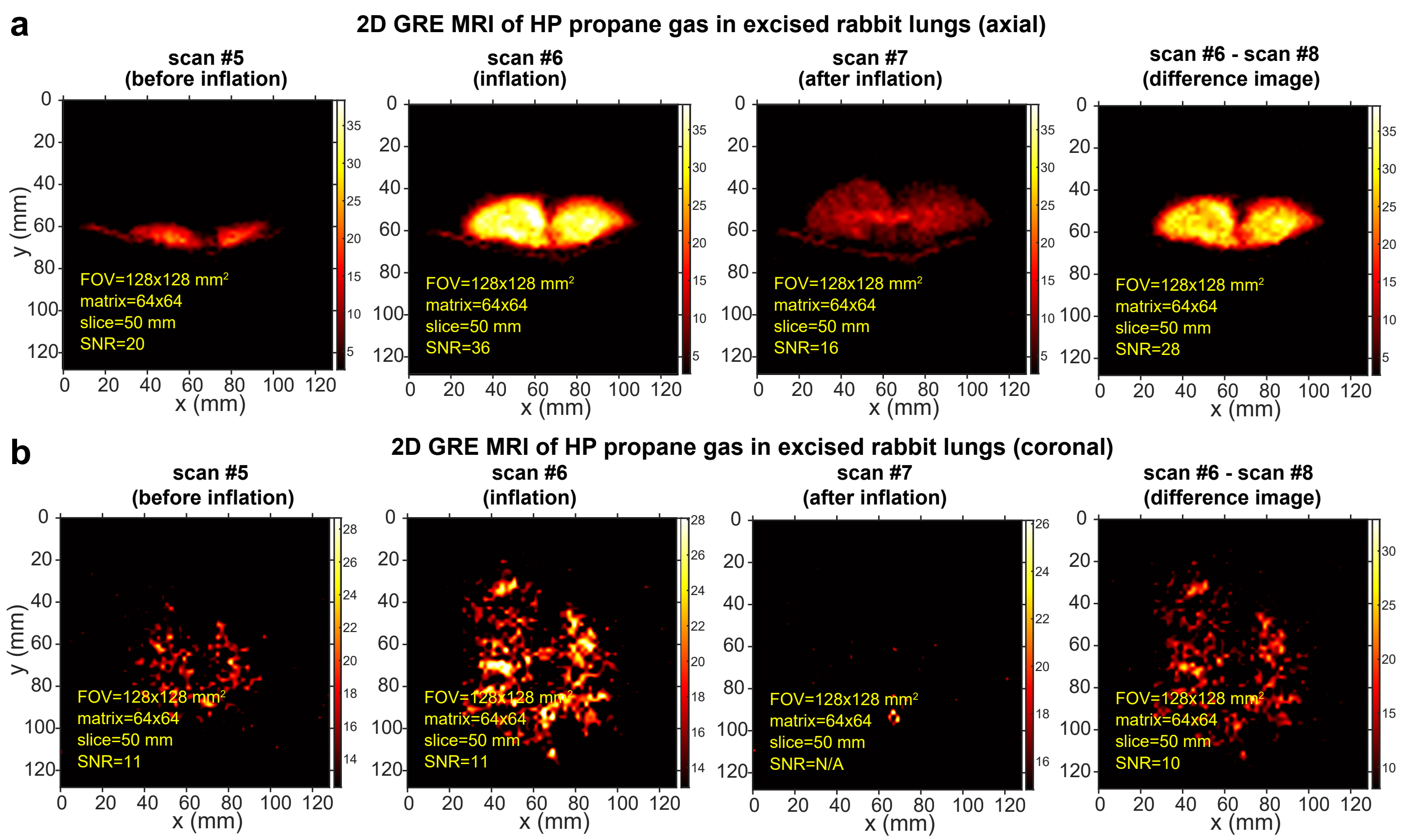

Figure S13. Sub-second slice-selective 2D GRE images of HP propane gas injected in excised rabbit lungs acquired by utilizing a 0.35 T MRI scanner and knee RF coil. a) Axial projection of the excised rabbit lungs recorded before inflation (scan #5), during inflation (scan #6), and after inflation (scan #7) with 0.94 s temporal resolution. b) Corresponding scans from coronal projection. The difference image for both axial and coronal projections were obtained as the differences of scan #6 and scan #8, because of the residual HP signal presence in after inflation scan #7 (also shown in **Figures S14** and **S15**). All the images from both axial and coronal projections were acquired with a 128×128 mm^2 FOV, slice thickness of 50 mm, 30° slice-selective RF excitation pulse, 64×64 imaging matrix, and post-processing image interpolation to 768×768 pixels.

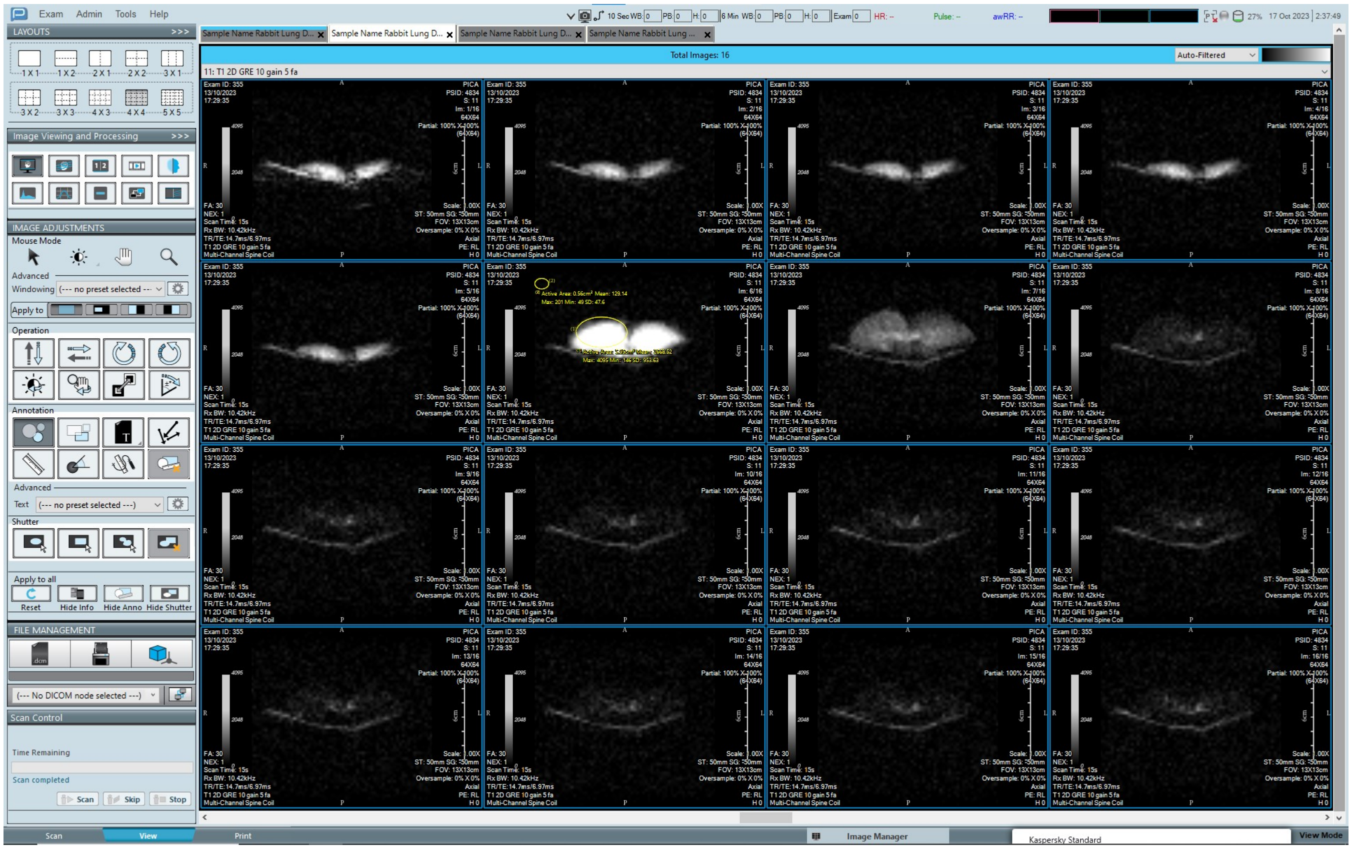

Figure S14. Screenshot of images processed by the MRI scanner software during the experiment of **Figure S13a** (axial projection, 2×2 mm^2 pixel size) without any data post-processing.

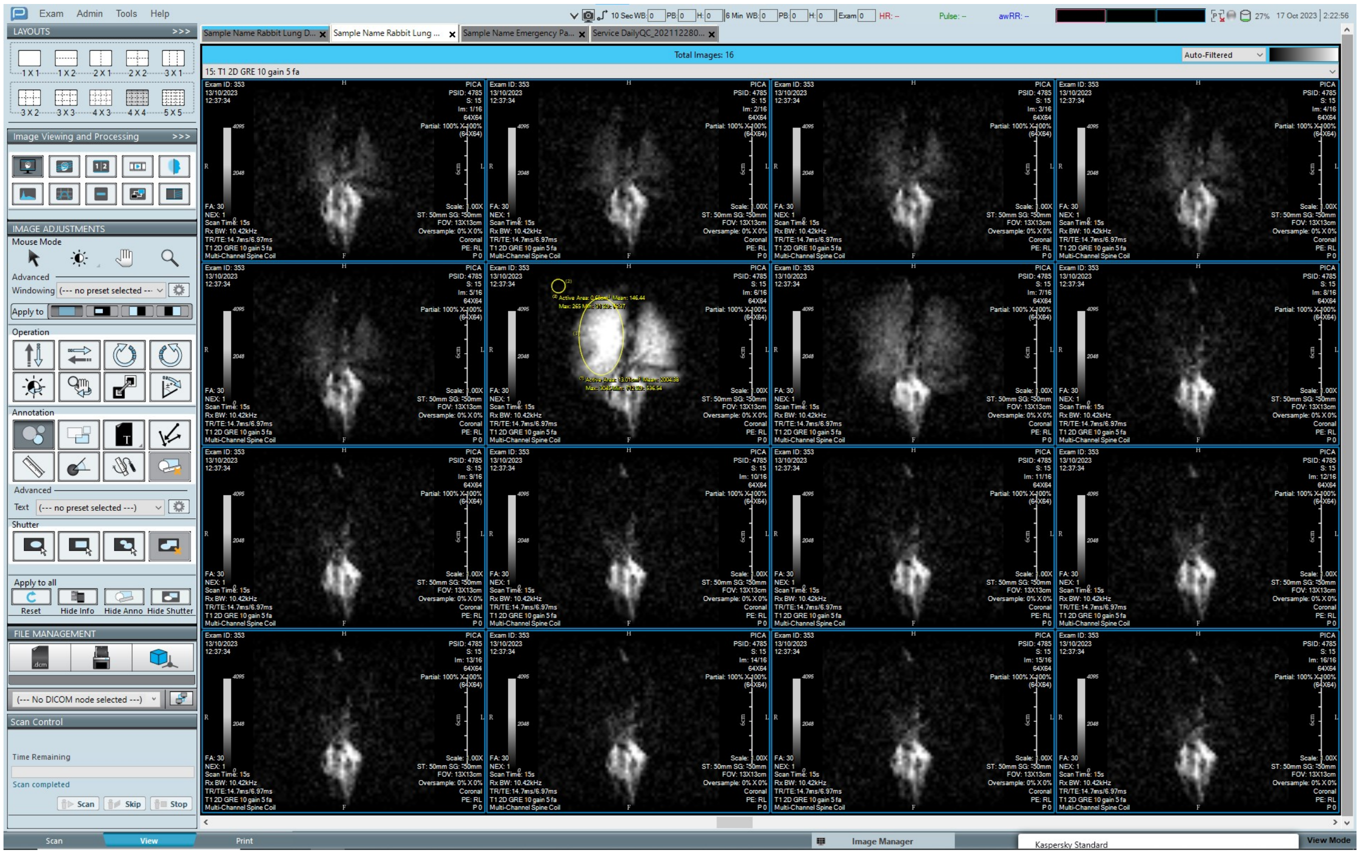

Figure S15. Screenshot of images processed by the MRI scanner software during the experiment of **Figure S13b** (coronal projection, 2×2 mm^2 pixel size) without any data post-processing.

2d. HP propane MR images with 160×160 mm^2 FOV and 2.5×2.5 mm^2 pixel size

Experiment #1

Figure S16a and **S16b** present the axial and coronal projection MR images of the excised rabbit lungs using HP propane. The mean SNR of the images shown in **Figure S16a** was 25 in before inflation, 39 in during inflation, and 16 in after inflation images, respectively. In **Figure S16b**, the mean SNR is 21 in before inflation, 23 in during inflation, and 22 in after inflation images, respectively. The imaging parameters used in these experiments were: 16 slices (total 15 seconds), 64×64 imaging matrix, 160×160 mm^2 field of view (FOV), 30° slice-selective RF excitation pulse, spectral width 10.42 kHz, repetition time (TR) 14.7 ms, echo time (TE) 6.97 ms. The difference images were obtained as the differences of scan #7 and scan #9 for axial (as there was residual signal in the after inflation axial image #8) and scan #6 and scan #8 for coronal projections, respectively, which yielded mean SNR of 28 (axial) and 16 (coronal) in **Figure S16**. The 16 axial and coronal images processed with the MRI scanner software before any MATLAB data processing are shown in **Figure S17** and **Figure S18**, respectively.

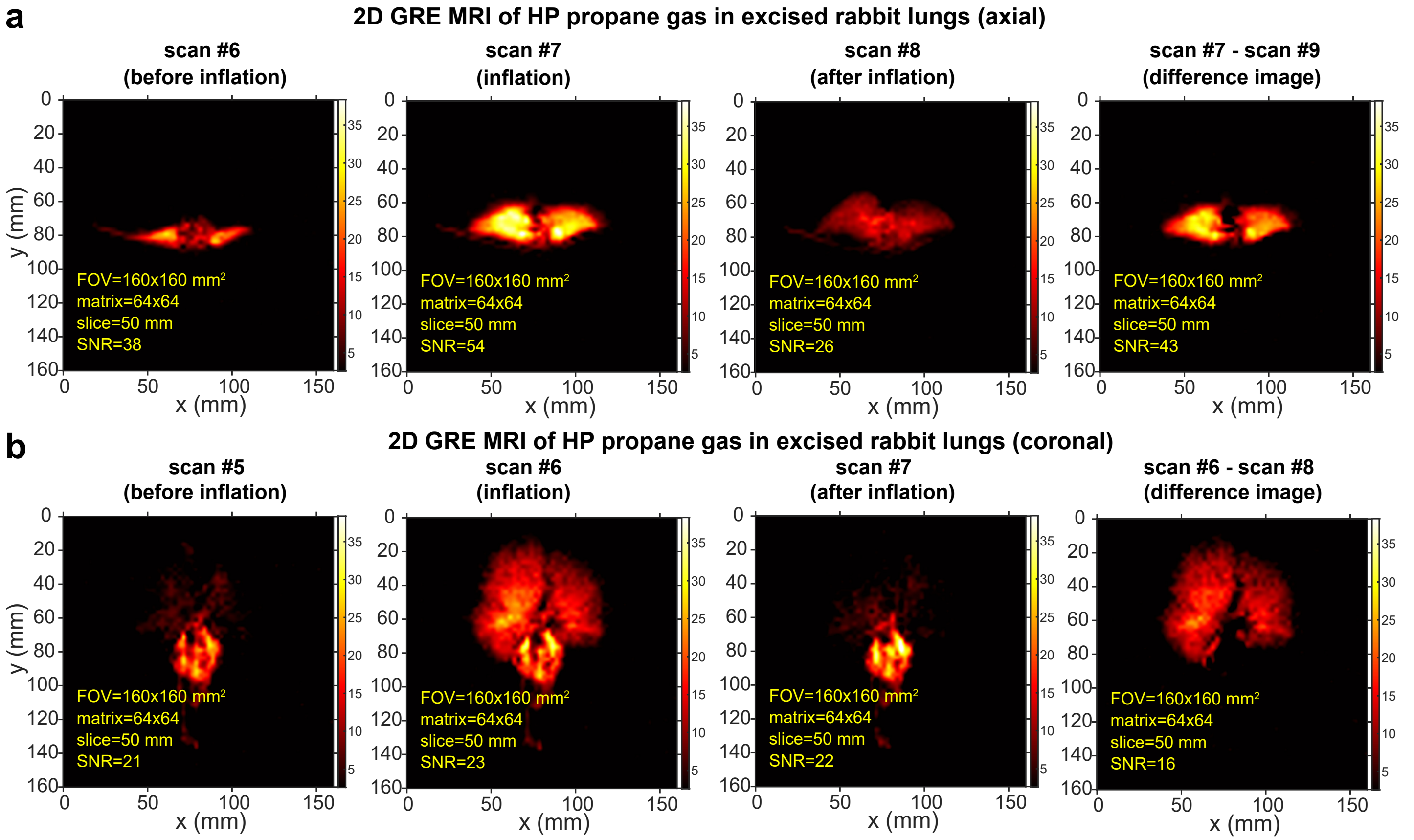

Figure S16. Sub-second slice-selective 2D GRE images of HP propane gas injected in excised rabbit lungs acquired by utilizing a 0.35 T MRI scanner and knee RF coil. a) Axial projection of the excised rabbit lungs recorded before inflation (scan #6), during inflation (scan #7), and after inflation (scan #8) with 0.94 s temporal resolution. b) Corresponding scans from coronal projection. The difference image for axial projection was obtained as the difference of scan #7 and scan #9, because of the residual HP signal presence in after inflation scan #8 (shown in **Figure S17**). For coronal projection difference image, the difference between scan #6 and scan #8 was taken (shown in **Figure S18**). All the images from both axial and coronal projections were acquired with a 160×160 mm^2 FOV, slice thickness of 50 mm, 30° slice-selective RF excitation pulse, 64×64 imaging matrix, and post-processing image interpolation to 768×768 pixels.

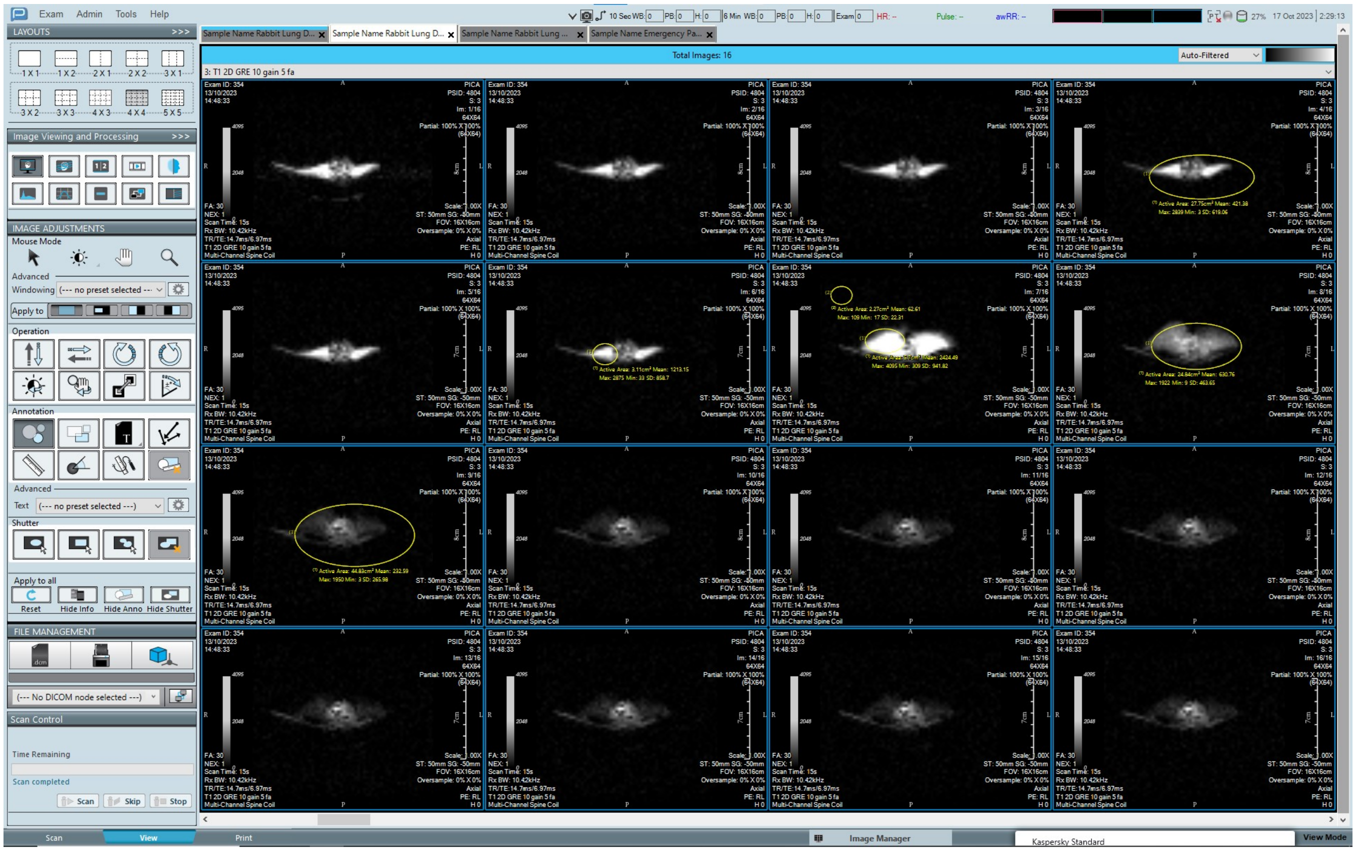

Figure S17. Screenshot of images processed by the MRI scanner software during the experiment of **Figure S16a** (axial projection, 2.5×2.5 mm^2 pixel size) without any data post-processing.

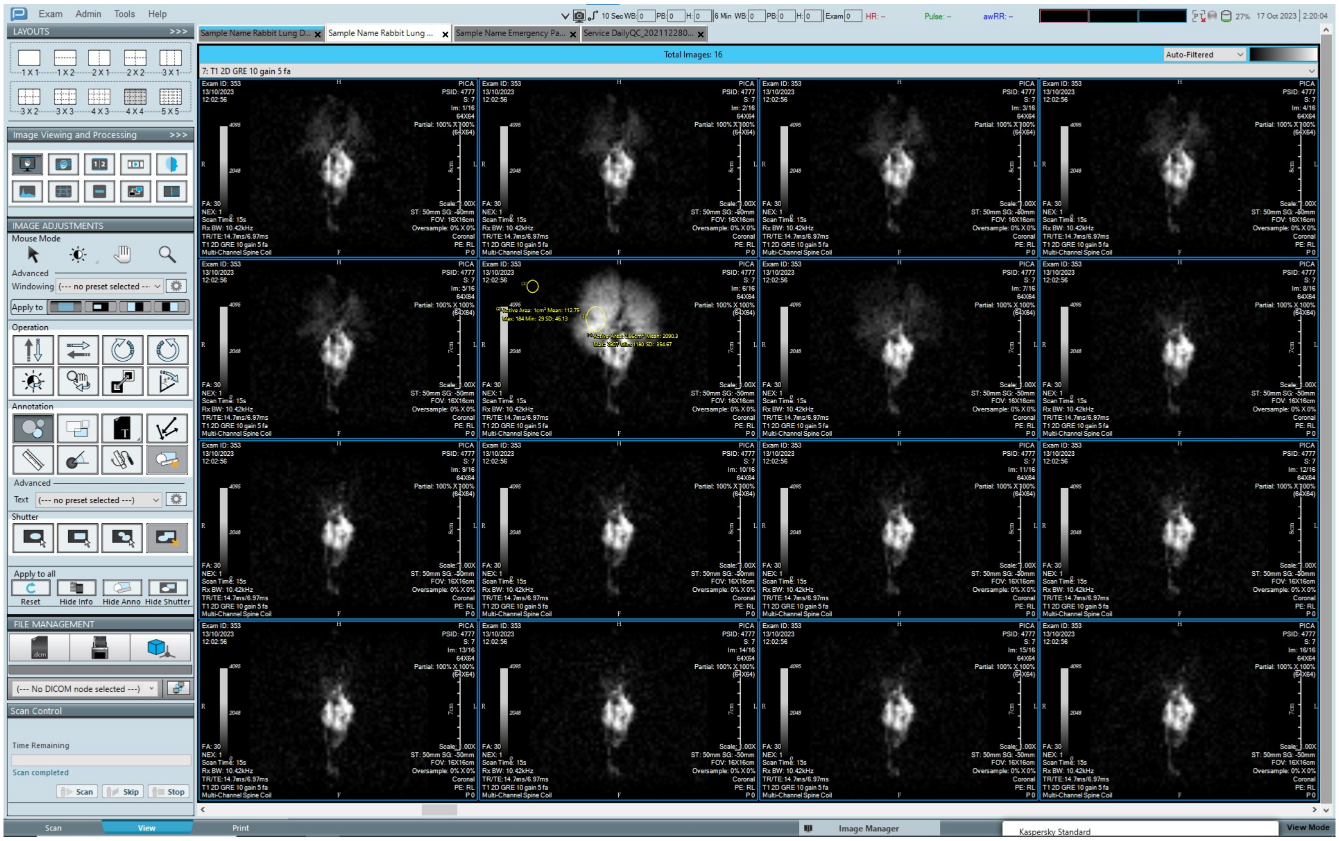

Figure S18. Screenshot of images processed by the MRI scanner software during the experiment of **Figure S16b** (coronal projection, 2.5×2.5 mm^2 pixel size) without any data post-processing.

Experiment #2

Figure S19a and **S19b** present the axial and coronal projection MR images of the excised rabbit lungs using HP propane gas. The mean SNR of the images shown in **Figure S19a** was 33 in before inflation, 34 in during inflation, and 23 in after inflation images, respectively, compared to **Figure S16a** mean SNR of 25, 39, and 16 in the corresponding images. In **Figure S19b**, the mean SNR is 21 in before inflation, 22 in during inflation, and 25 in after inflation images, respectively, compared to 21, 23, and 22 in the corresponding images in **Figure S16**. The imaging parameters used in these experiments were: 16 slices (total 15 seconds), 64×64 imaging matrix, 160×160 mm^2 field of view (FOV), 30° slice-selective RF excitation pulse, spectral width 10.42 kHz, repetition time (TR) 14.7 ms, echo time (TE) 6.97 ms. The difference images were obtained as the differences of scan #6 and scan #8 for axial (as there was residual signal in the after inflation axial image #7) and scan #6 and scan #8 for coronal projections, respectively, which yielded mean SNR of 23 (axial) and 15 (coronal) in **Figure S19** compared to 28 (axial) and 22 (coronal) in **Figure S16**, clearly shown good back-to-back scan reproducibility. The 16 axial and coronal images processed with the MRI scanner software before any MATLAB data processing are shown in **Figure S20** and **Figure S21**, respectively.

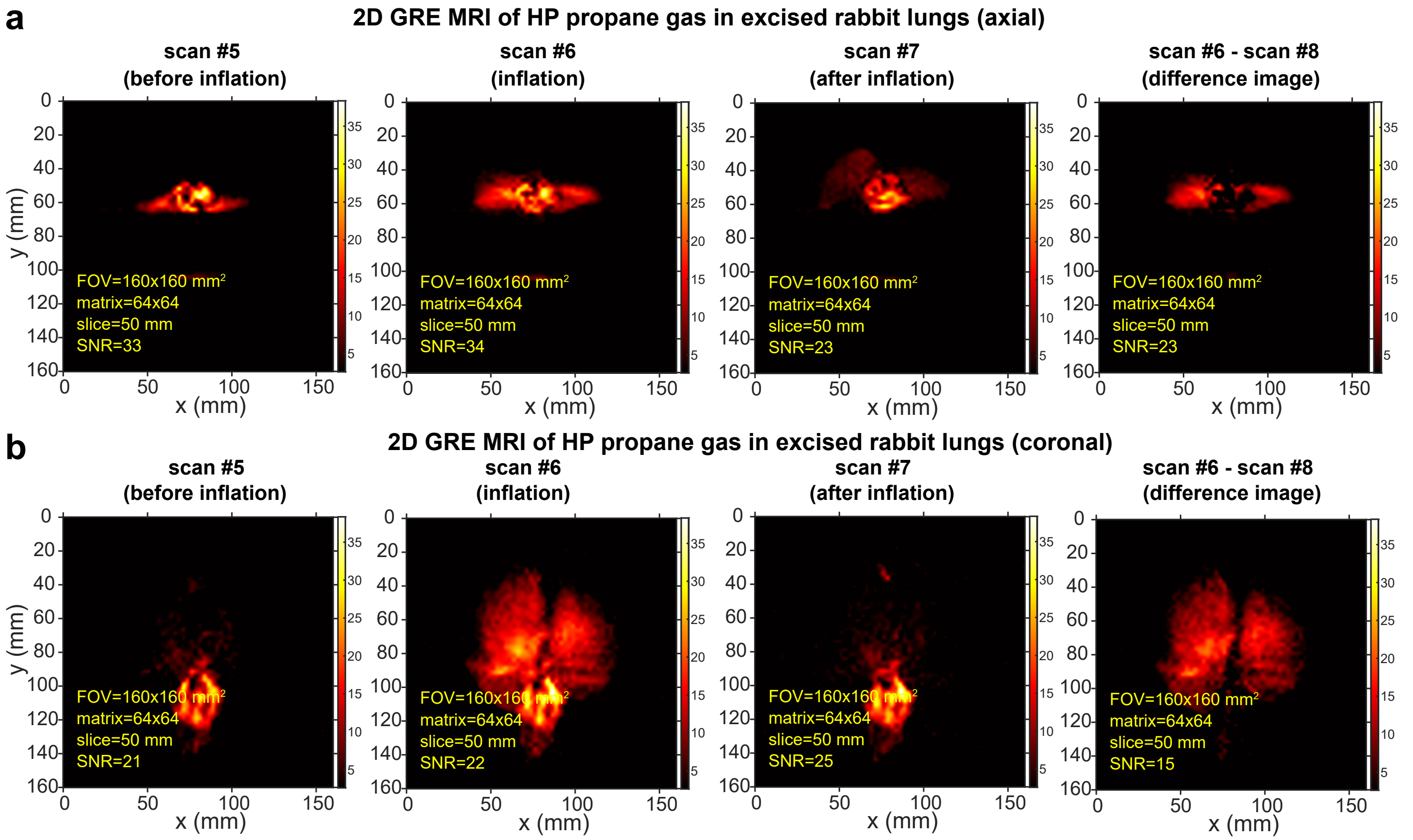

Figure S19. Sub-second slice-selective 2D GRE images of HP propane gas injected in excised rabbit lungs acquired by utilizing a 0.35 T MRI scanner and knee RF coil. a) Axial projection of the excised rabbit lungs recorded before inflation (scan #5), during inflation (scan #6), and after inflation (scan #7) with 0.94 s temporal resolution. b) Corresponding scans from coronal projection. The difference image for axial projection was obtained as the difference of scan #6 and scan #8, because of the residual HP signal presence in after inflation scan #7 (shown in **Figure S20**). For coronal projection difference image, the difference between scan #6 and scan #7 was taken (shown in **Figure S21**). All the images from both axial and coronal projections were acquired with a 160×160 mm^2 FOV, slice thickness of 50 mm, 30° slice-selective RF excitation pulse, 64×64 imaging matrix, and post-processing image interpolation to 768×768 pixels.

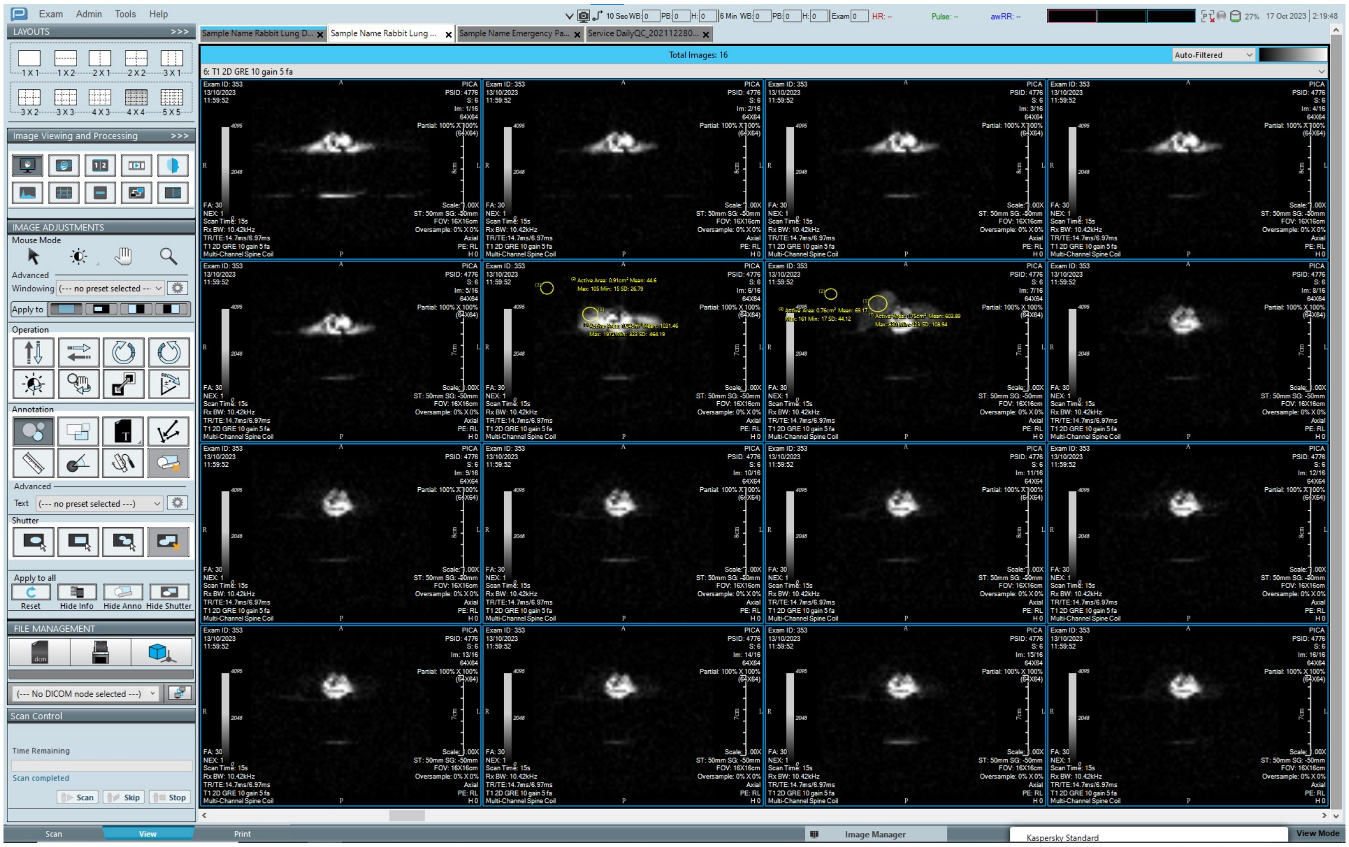

Figure S20. Screenshot of images processed by the MRI scanner software during the experiment of **Figure S19a** (axial projection, 2.5×2.5 mm^2 pixel size) without any data post-processing.

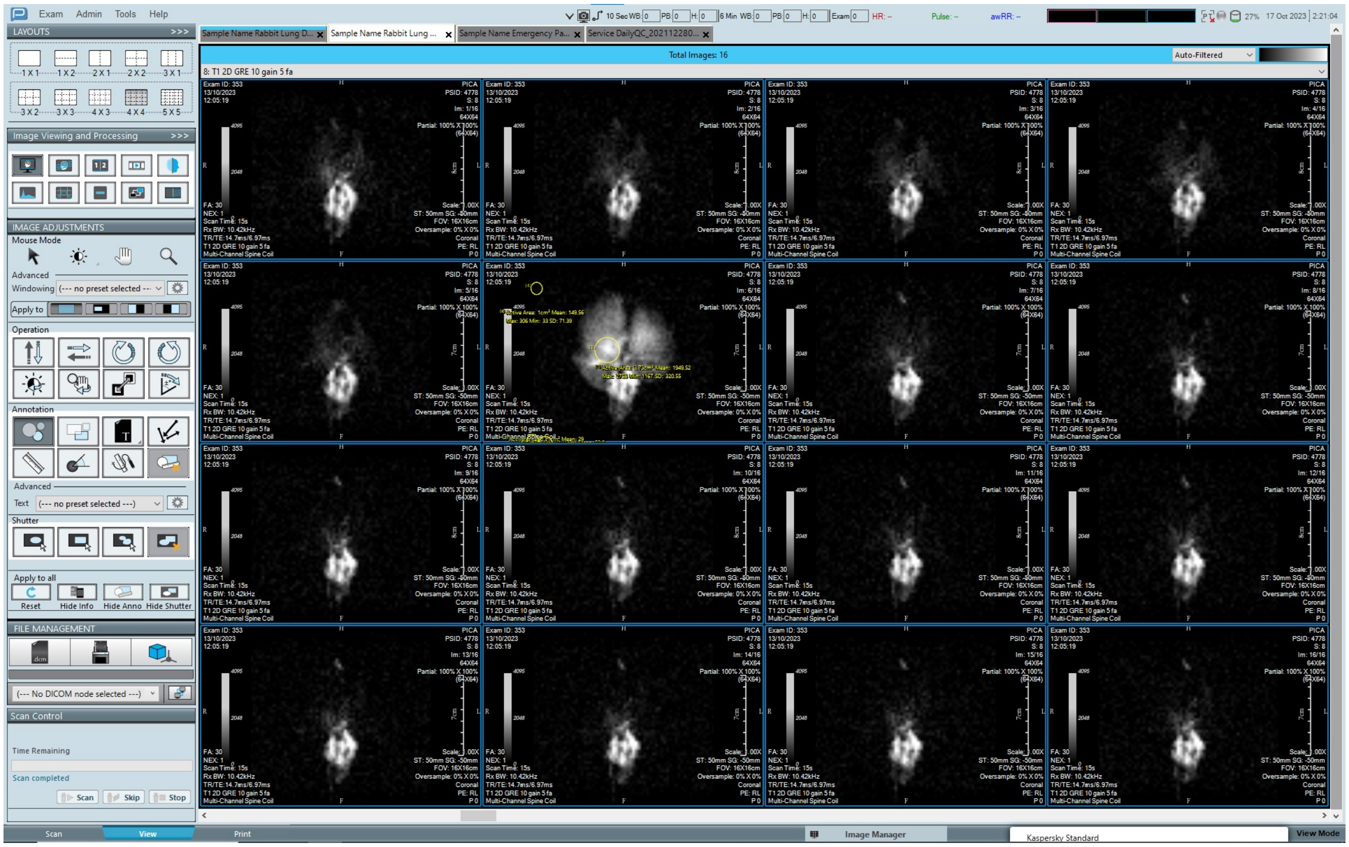

Figure S21. Screenshot of images processed by the MRI scanner software during the experiment of **Figure S19b** (coronal projection, 2.5×2.5 mm^2 pixel size) without any data post-processing.

3. Screenshots of HP propane MR images displayed in the main text Figure 2

During the MR imaging of the excised rabbit lungs the acquired raw data was also processed by the MRI scanner software. This scanner processing allows visual representation of acquired images. **Figure S22** and Figure S23 are corresponding MRI scanner software screenshots of the imaging series presented in Figure 2a and Figure 2b, respectively. These screenshots also provide information about the imaging pulse sequence parameters that were used during the particular experiments. These parameters include but not limited to FOV, matrix size, TR/TE values, scan time, scan type etc. Additionally, these screenshots guided the advanced image processing that was later performed utilizing MATLAB.

In this section we provide the raw images of all 16 repetition scans from the experiments presented in **Figure 2** of the main text.

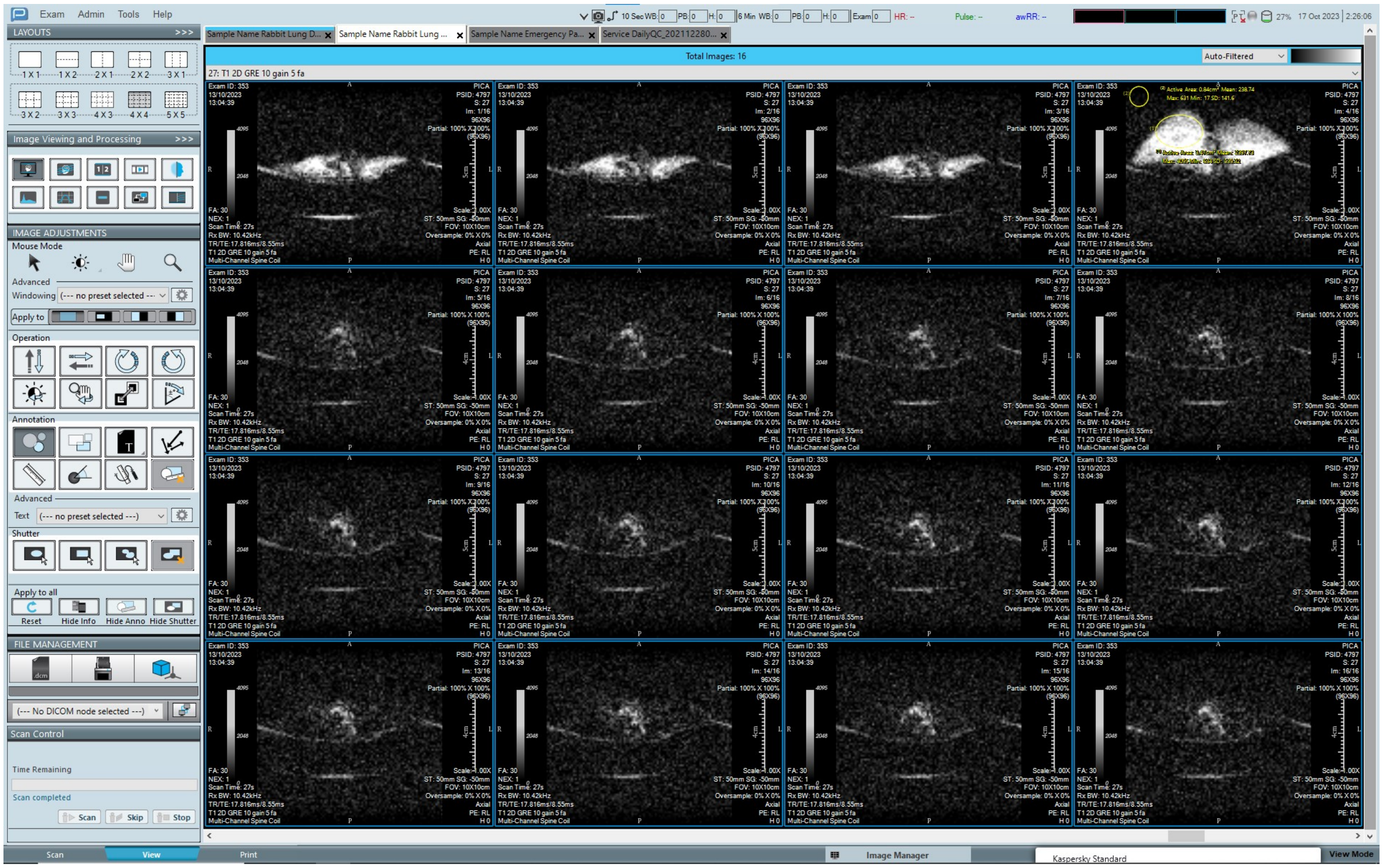

Figure S22. Screenshot of images processed by the MRI scanner software during the experiment of main text **Figure 2a** (axial projection, 1×1 mm^2 pixel size) without any data post-processing.

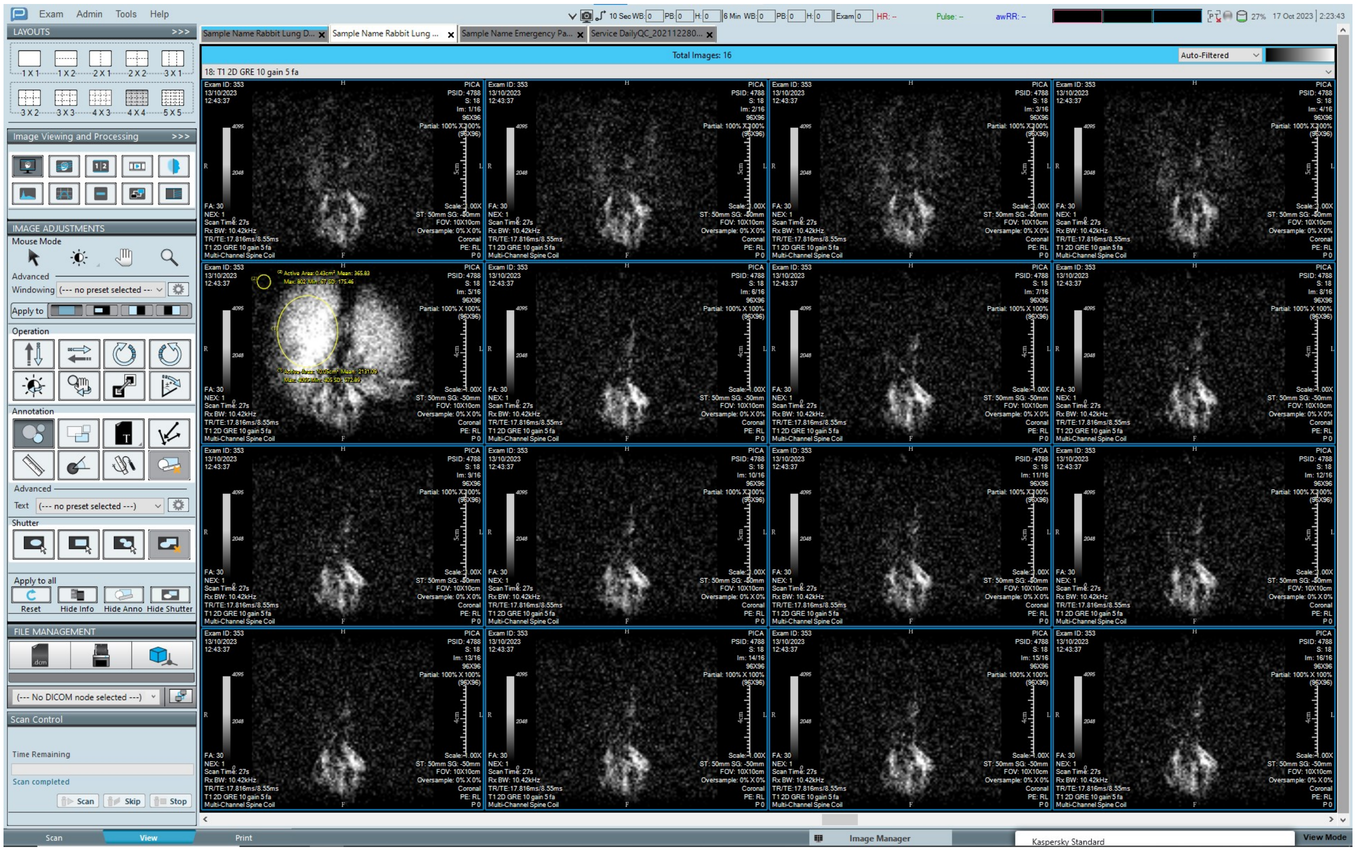

Figure S23. Screenshot of images processed by the MRI scanner software during the experiment of main text **Figure 2b** (coronal projection, 1×1 mm^2 pixel size) without any data post-processing.

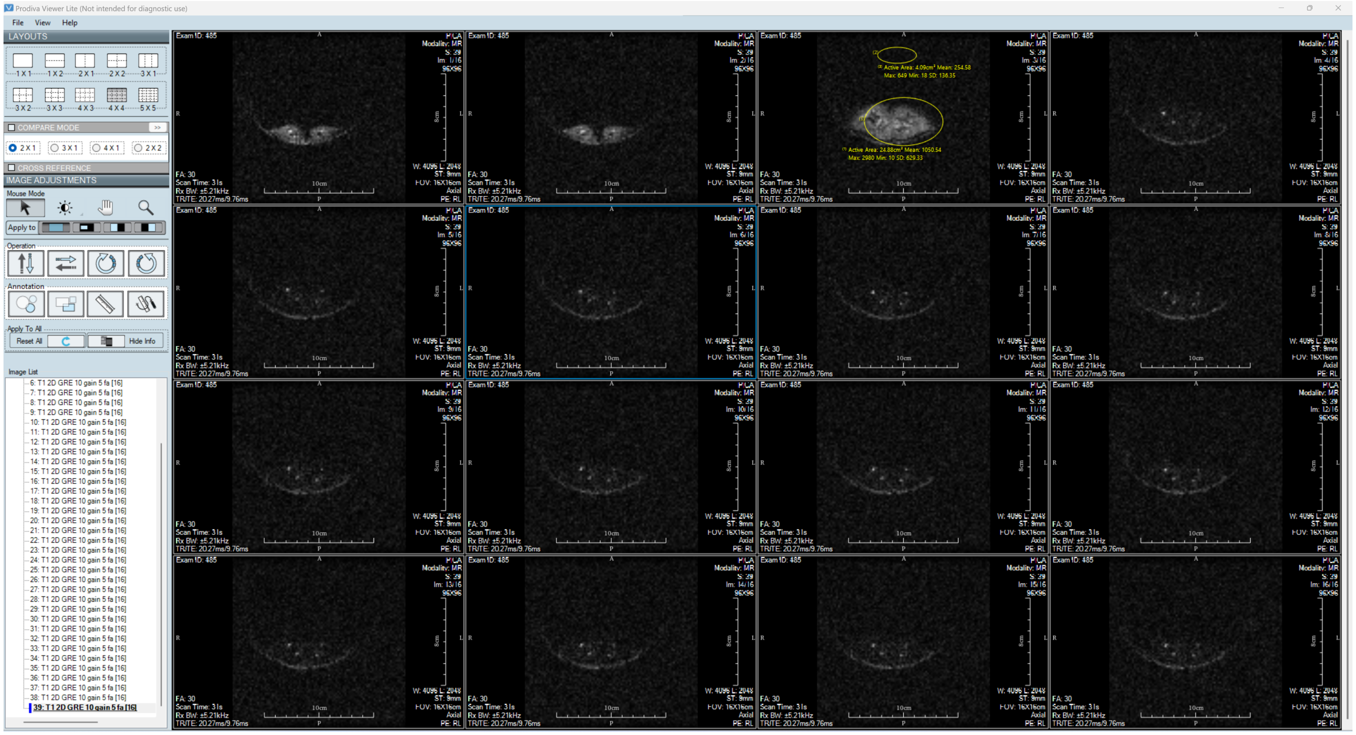

Figure S24. Screenshot of images processed by the MRI scanner software during the experiment of main text **Figure 3** (axial projection, 1.7×1.7 mm^2 pixel size) without any data post-processing.

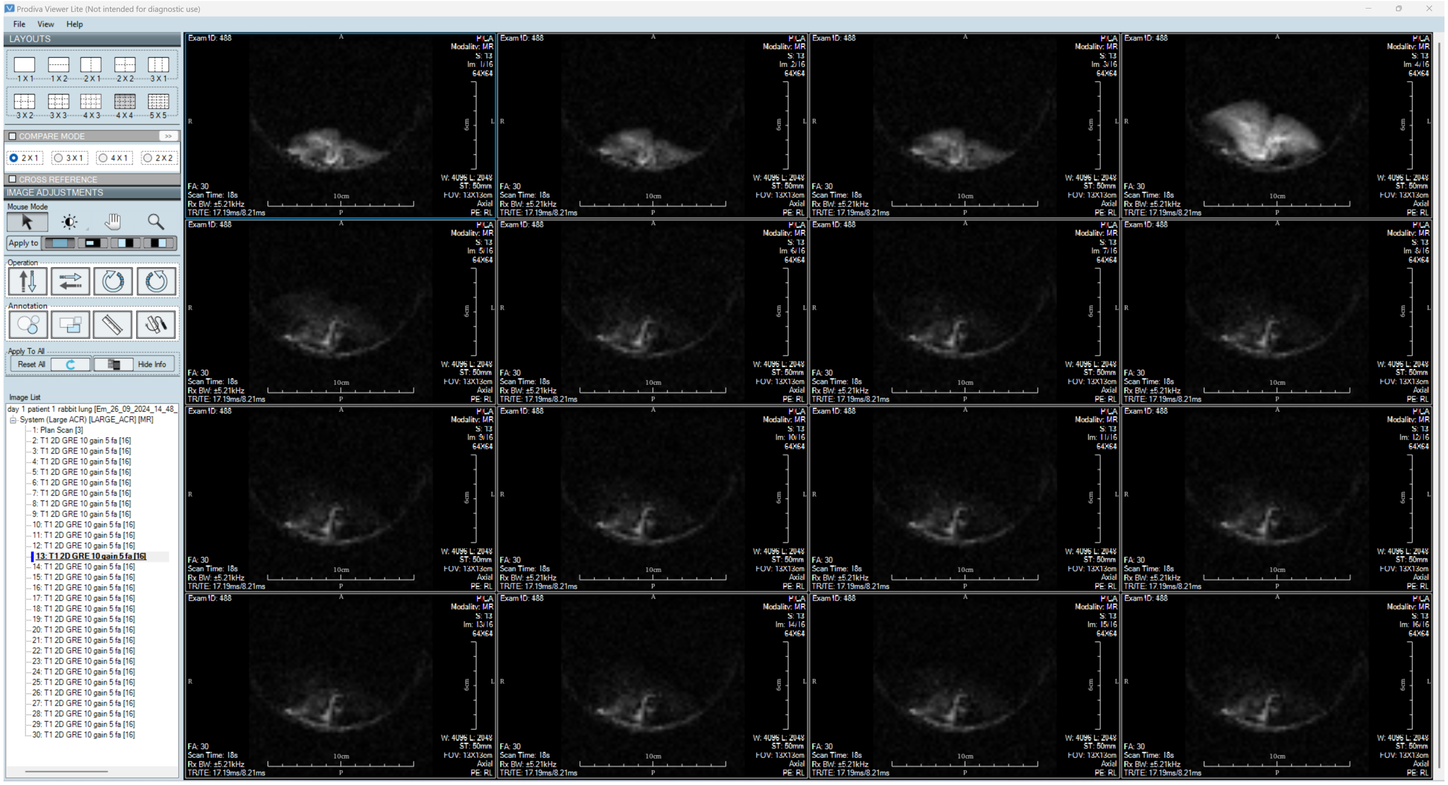

Figure S25. Screenshot of images processed by the MRI scanner software during the experiment of main text **Figure 4a** (axial projection, 2×2 mm^2 pixel size) without any data post-processing. HP propane gas injection was employed as seen in scan #4.

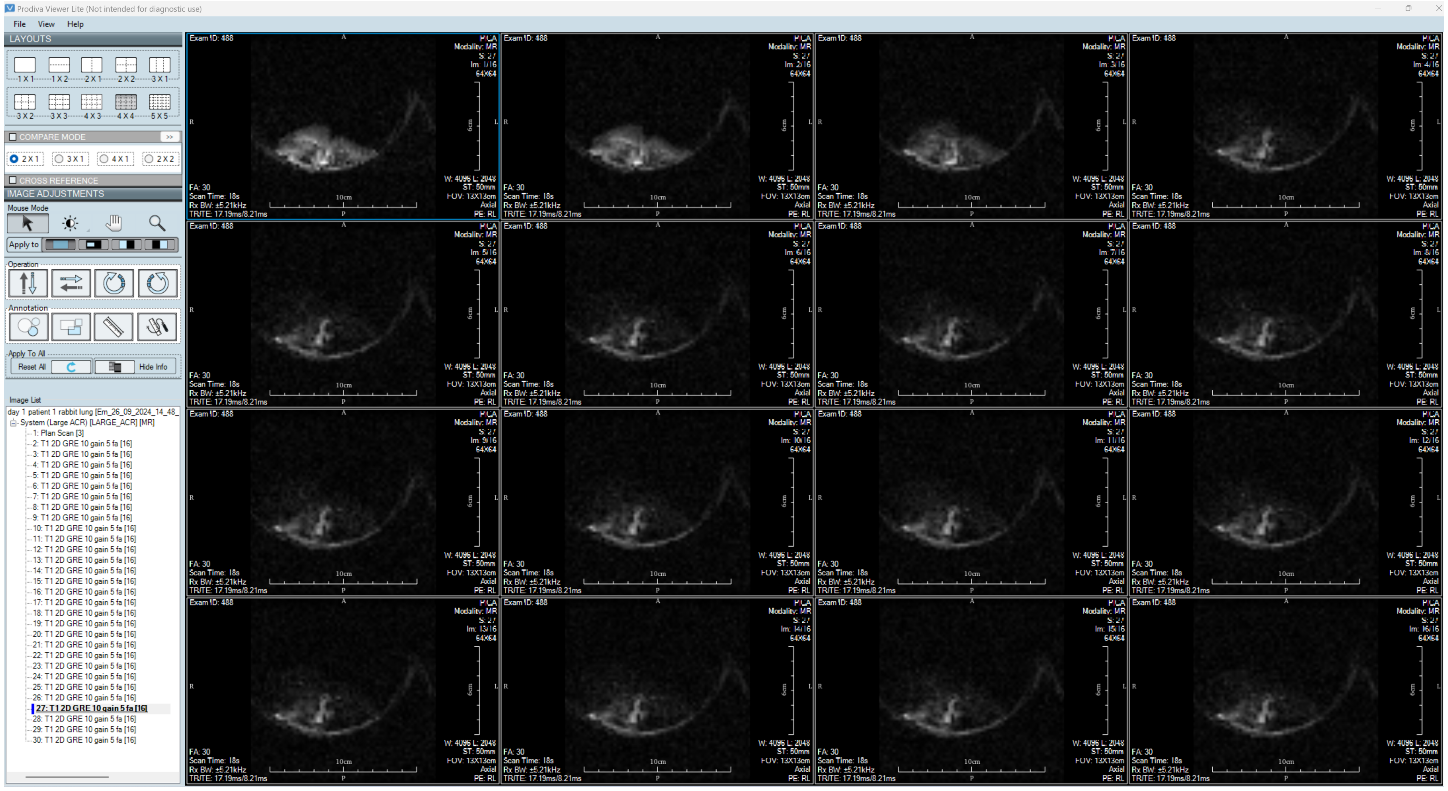

Figure S26. Screenshot of images processed by the MRI scanner software during the experiment of main text **Figure 4b** (axial projection, 2×2 mm^2 pixel size) without any data post-processing. Control inert nitrogen gas injection was employed.

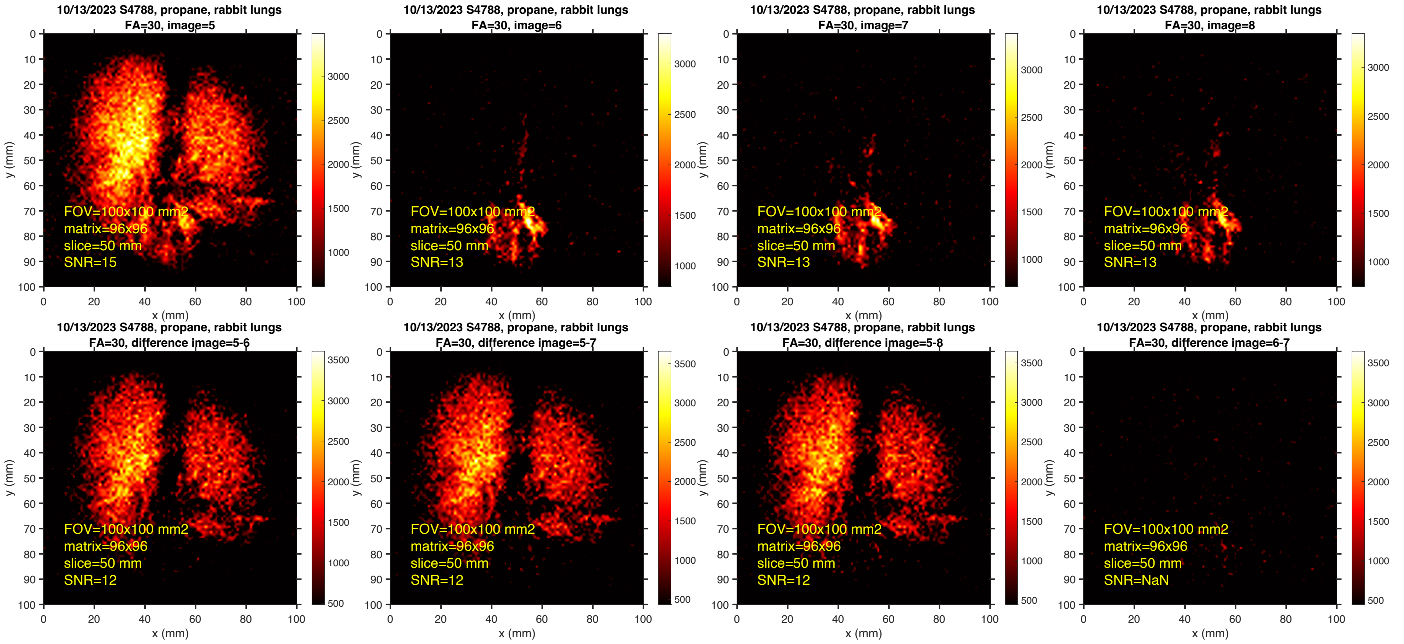

Figure S27. Additional supporting images for **Figure 2b**. (top row): imaging series extended showing image #5 (shown in **Figure 2b**), image #6 (shown in **Figure 2b**), image #7 (not shown in **Figure 2b**), and image #8 (not shown in **Figure 2b**); (bottom row): difference image #6 subtracted from #5 (shown in **Figure 2b**), difference image #7 subtracted from #5 (not shown in **Figure 2b**), difference image #8 subtracted from #5 (not shown in **Figure 2b**), difference image #7 subtracted from #6 (not shown in **Figure 2b**).

4. MATLAB image processing

This section describes the image processing steps applied to DICOM data in this study. Each DICOM file represents a single image slice with varying sizes like 96×96 or 64×64. All pulse sequences included 16 repeat scans, resulting in 16 DICOM files per experiment.

I. **Reading DICOM Files:** The "dicomread" function in MATLAB reads all DICOM slices for a single experiment.

II. **Image Display:** The raw images are displayed with their corresponding field of view (FOV), such as 100×100, 128×128, or 160×160.

III. **Subtraction Image:** The difference between an inflated lung image and a post-inflation image (without residual HP signal) creates the difference image.

IV. **Mean Signal-to-Noise Ratio (SNR):** All image pixels are analyzed for their intensities. First, the region of the image (8×8 pixels in size), where no signal is seen by a user is selected to compute the RMS noise value. This RMS noise value is employed for identifying pixels that contain a signal. When a pixel intensity exceeds a threshold value (computed based on the RMS noise value), the pixel signal is added to the overall calculation of SNR (specifically, the signal is added to the total sum of all signal values from all pixels exceeded the threshold value); in case if the pixel intensity is below the threshold value, it is discarded from the SNR calculation. Next, the mean signal is computed by finding the mean values of all the pixels exceeding the threshold value. Finally, the SNR is computed by dividing the mean signal value by the value of RMS noise. This approach mitigates the need for the region of interest (ROI) selection by an end user.

V. **Image Resizing:** For better visualization, both the raw and difference images are resized to 768×768 matrices. This resizing is applied to all images in the study.

The following MATLAB code #1 was used for data processing for image visualization and SNR calculation (example is provided for the data shown in **Figure 2a**).

```
cla;
clear;
close all;
% parameters to ENTER
FOV=100;                                % enter FOV from the image information
matrix=96;                              % enter matrix size from the image information
fa = 30;                                % flip angle value: will be used for display only
slice = 50;                                % slide thickness value: will be used for
display only
noise_threshold = 10;                     % thresholding for image presentation: everything
less than RMS noise * this value will be dropped
exp_number = 353;                          % experiment number: : will be used for display
only
date_value = '10/13/2023';              % date: : will be used for display only
gas_value = 'propane';                  % type of gas (e.g., propane): : will be used for
display only
subject = 'rabbit lungs';                % type of gas (e.g., rabbit lungs): : will be used
for display only
x_position = 8;                       % x-position for the output of key imaging parameters
y_position = 80;                      % y-position for the output of key imaging parameters
%computed parameters
slice_string = int2str(slice);            % converts slice thickness value to a string
fa_string = int2str(fa);                 % converts FA value to a string
fa_string = append('FA=',fa_string);                  % finished FA string composition
matrix_string = int2str(matrix);        % converts matrix size to a string
fov_string = int2str(FOV);              % converts FOV value to a string
```

```
exp_string = int2str(exp_number);       % converts exp number to a string
exp_string = append('S',exp_string);       % creates S number as a string
date_string = date_value;       % converts date into a string
gas_string = gas_value;       % converts gas type into a string
subject_string = subject;       % converts subject type into a string


% Specify the paths of the DICOM files
dicomPaths = {
'I0000001.dcm',
'I0000002.dcm',
'I0000003.dcm',
'I0000004.dcm',
'I0000005.dcm',
'I0000006.dcm',
'I0000007.dcm',
'I0000008.dcm',
'I0000009.dcm',
'I0000009.dcm',
'I0000010.dcm',
'I0000011.dcm',
'I0000012.dcm',
'I0000013.dcm',
'I0000014.dcm',
'I0000015.dcm',
'I0000016.dcm',
};

% Read the DICOM files
dicomFiles = cell(16, 1);
for i = 1:16
    dicomFiles{i} = double(dicomread(dicomPaths{i}));
end

% reate a subplot to plot the resized image (one at a time)
figure;

for i = 1:16
    % Resize the image to 768x768
    counter = 0;        % resets the counter for the number of noise calculation
    signalsum = 0;      % resets the signal pixel count for noise calculation
    original_image{i}=dicomFiles{i};    % loads the images
    resized_image = imresize(double(dicomFiles{i}), [768, 768]);     % loads the images
    % compute noise:
    noise_image = original_image{i}(4:11, 10:17);    % selects the region for noise
computations
    offset = mean(noise_image);                     % computing the mean for each column
of the image matrix
    offset1 = mean(offset);                         % computing the mean for all elements
of the image matrix
    noise_diff = noise_image-offset1;
    noiserms = (rms(noise_image-offset1));         % computing RMS noise of each column
of the noise matrix after subtracting the mean value
    noiserms1 = (rms(noiserms));                    % computing RMS noise of the image
matrix
    noise_counter = noiserms1*noise_threshold;     % computing noise value for
thresholding using the factor noise_threshold
    % compute average SNR using noise thresholding
for k = 1:matrix
for j = 1:matrix
```

```
    if original_image{i}(k, j) > noise_counter          % compares pixel value to noise
threshold
        signalsum = signalsum + original_image{i}(k, j)/noiserms1;  % if the pixel value
is greater than the threshold value, it is added to the total SNR sum
        counter = counter +1;                           % counts the number of pixels
that exceeded the noise threshold value
    end
end
end
    SNR_average = signalsum/counter;                % computes the average SNR of all
signal pixels that have exceeded the noise threshold value
    snr_string = int2str(SNR_average);            % converts SNR value to a string
    %image plotting
    imshow(resized_image, [], 'XData', [0, FOV], 'YData', [0, FOV]);        %
broadcasting the image in the figure dispplay (one at a time)
    YourText = sprintf(append('FOV=',fov_string,'x',fov_string,' mm2
\nmatrix=',matrix_string,'x',matrix_string,'\nslice=',slice_string,'
mm\nSNR=',snr_string));  % yellow text
    hText = text(x_position,y_position,YourText,'Color',[1 1 0],'FontSize',18); %yellow
text position and color %! changes second number to 25(from 20) as my texts were popping
out of the figure frame at the top
    caxis([noise_counter/2, 4095-noise_counter/2]);  % setting the threshold for image
visualization
    ax = gca;   %standard axes
    axis on;    % axes to be displays (ON)
    i_string = int2str(i);  % creates string value of i (the loop counter)
    title_name = append(date_string,' ',exp_string,', ',gas_string,',
',subject_string,'\n',fa_string,', image=',i_string);
    title(sprintf(title_name));             % title statement using title_name file
    ax.FontSize = 14;                       % font size of the axes
    ax.TickDir = 'out';                     % ticks are out not in
    ax.TickLength = [0.015 0.015];          % ticks' length
    ax.XAxis.MajorTickChild.LineWidth = 2;  % x axis tickmark thickness = 2
    ax.YAxis.MajorTickChild.LineWidth = 2;  % y axis tickmark thickness = 2
    xlabel('x (mm)')        % x axis label
    ylabel('y (mm)')        % y axis label
    colorbar                % displays the color bar
    colormap(hot);          %  color mode: other good color modes are: 'gray' and 'jet'
    filename = append(exp_string,'_',i_string,'.pdf');
    %exportgraphics(gcf, sprintf(filename), 'ContentType', 'vector'); %!
    %line wont work on my mathlab. errors says unrecognized function or
    %variable 'exportgraphics'
    saveas(gcf, sprintf(filename)); %!line added to be able to save images generated
during analysis
end
```

The following MATLAB code #2 was used for data processing for image subtraction visualization and SNR calculation (example is provided for the data shown in **Figure 2a**).

```
cla;
clear;
close all;
% parameters to ENTER
FOV=100;                                %! enter FOV from the image information
matrix=96;                              % enter matrix size from the image information
fa = 30;                                % flip angle value: will be used for display only
slice = 50;                                % slide thickness value: will be used for
display only
```

```
noise_threshold = 10;                    % thresholding for image presentation: everything
less than RMS noise * this value will be dropped
subt_image_number = 5;                   % image number to be subtracted
exp_number = 353;                         % experiment number: : will be used for display
only
date_value = '10/13/2023';              % date: : will be used for display only
gas_value = 'propane';                  % type of gas (e.g., propane): : will be used for
display only
subject = 'rabbit lungs';                % type of gas (e.g., rabbit lungs): : will be used
for display only
x_position = 8;                        % x-position for the output of key imaging parameters
y_position = 80;                       % y-position for the output of key imaging parameters
%computed parameters
slice_string = int2str(slice);           % converts slice thickness value to a string
fa_string = int2str(fa);                 % converts FA value to a string
fa_string = append('FA=',fa_string);                % finished FA string composition
matrix_string = int2str(matrix);        % converts matrix size to a string
fov_string = int2str(FOV);              % converts FOV value to a string
exp_string = int2str(exp_number);        % converts exp number to a string
exp_string = append('S',exp_string);        % creates S number as a string
subt_string = int2str(subt_image_number);       % converts # of image to be subtracted
into a string
date_string = date_value;       % converts date into a string
gas_string = gas_value;       % converts gas type into a string
subject_string = subject;       % converts subject type into a string

% Specify the paths of the DICOM files
dicomPaths = {
'I0000001.dcm',
'I0000002.dcm',
'I0000003.dcm',
'I0000004.dcm',
'I0000005.dcm',
'I0000006.dcm',
'I0000007.dcm',
'I0000008.dcm',
'I0000009.dcm',
'I0000010.dcm',
'I0000011.dcm',
'I0000012.dcm',
'I0000013.dcm',
'I0000014.dcm',
'I0000015.dcm',
'I0000016.dcm',
};

% Read the DICOM files
dicomFiles = cell(16, 1);
for i = 1:16
    dicomFiles{i} = double(dicomread(dicomPaths{i}));
end

% Create scan #99 using the scan to be subtracted
for i = subt_image_number:subt_image_number
    for x = 1:matrix
    for y = 1:matrix
        dicomFiles{99}(x, y) = dicomFiles{i}(x, y);      % assigning the matrix number
and its elements
    end
    end
```

```
end
% Perfoming the background subtraction
for i = 1:16
    for x = 1:matrix
    for y = 1:matrix
        dicomFiles_diff{i}(x, y) = dicomFiles{i}(x, y) - dicomFiles{99}(x, y);      %
making the subtraction
    end
    end
end

% Create a subplot to plot the resized image (one at a time)
figure;

for i = 1:16
    % Resize the image to 768x768
    counter = 0;        % resets the counter for the number of noise calculation
    signalsum = 0;      % resets the signal pixel count for noise calculation
    original_image{i}=dicomFiles_diff{i};   % loads the images
    resized_image = imresize(double(dicomFiles_diff{i}), [768, 768]); % loads the images
    % compute noise:
    noise_image = original_image{i}(4:11, 10:17);    % selects the region for noise
computations
    offset = mean(noise_image);                     % computing the mean for each column
of the image matrix
    offset1 = mean(offset);                         % computing the mean for all elements
of the image matrix
    noise_diff = noise_image-offset1;
    noiserms = (rms(noise_image-offset1));         % computing RMS noise of each column
of the noise matrix after subtracting the mean value
    noiserms1 = (rms(noiserms));                    % computing RMS noise of the image
matrix
    noise_counter = noiserms1*noise_threshold;     % computing noise value for
thresholding using the factor noise_threshold
    % compute average SNR using noise thresholding
for k = 1:matrix
for j = 1:matrix
    if original_image{i}(k, j) > noise_counter          % compares pixel value to noise
threshold
        signalsum = signalsum + original_image{i}(k, j)/noiserms1;  % if the pixel value
is greater than the threshold value, it is added to the total SNR sum
        counter = counter +1;                           % counts the number of pixels
that exceeded the noise threshold value
    end
end
end
    SNR_average = signalsum/counter;                % computes the average SNR of all
signal pixels that have exceeded the noise threshold value
    snr_string = int2str(SNR_average);            % converts SNR value to a string
    %%
    %image plotting
    imshow(resized_image, [], 'XData', [0, FOV], 'YData', [0, FOV]);            %
broadcasting the image in the figure dispplay (one at a time)
    YourText = sprintf(append('FOV=',fov_string,'x',fov_string,' mm2
\nmatrix=',matrix_string,'x',matrix_string,'\nslice=',slice_string,'
mm\nSNR=',snr_string)); % yellow text
    hText = text(x_position,y_position,YourText,'Color',[1 1 0],'FontSize',18); %yellow
text position and color %! changes second number to 25(from 20) as my texts were popping
out of the figure frame at the top
```

```
    caxis([noise_counter/4, 4095-noise_counter/4]); % setting the threshold for image
visualization
    ax = gca;   %standard axes
    axis on;    % axes to be displays (ON)
    i_string = int2str(i);  % creates string value of i (the loop counter)
    title_name = append(date_string,' ',exp_string,', ',gas_string,',
',subject_string,'\n',fa_string,', difference image=',i_string,'-',subt_string);
    title(sprintf(title_name));           % title statement using title_name file
    ax.FontSize = 14;                     % font size of the axes
    ax.TickDir = 'out';                   % ticks are out not in
    ax.TickLength = [0.015 0.015];        % ticks' length
    ax.XAxis.MajorTickChild.LineWidth = 2;  % x axis tickmark thickness = 2
    ax.YAxis.MajorTickChild.LineWidth = 2;  % y axis tickmark thickness = 2
    xlabel('x (mm)')         % x axis label
    ylabel('y (mm)')         % y axis label
    colorbar                 % displays the color bar
    colormap(hot);           %  color mode: other good color modes are: 'gray' and 'jet'
    filename = append(exp_string,'_difference_',i_string,'-',subt_string,'.pdf');
    %exportgraphics(gcf, sprintf(filename), 'ContentType', 'vector'); %!
    %line wont work on my mathlab. errors says unrecognized function or
    %variable 'exportgraphics'
    saveas(gcf, sprintf(filename)); %!line added to be able to save images generated
during analysis
end
```

5. References Used in Electronic Supporting Information (ESI)

1. N. M. Ariyasingha, M. R. H. Chowdhury, A. Samoilenko, O. G. Salnikov, N. V. Chukanov, L. M. Kovtunova, V. I. Bukhtiyarov, Z. Shi, K. Luo, S. Tan, I. V. Koptyug, B. M. Goodson and E. Y. Chekmenev, *Chem. Eur. J.*, 2024, **30**, e202304071.
